# Trait Mapping Utilizing a Newly Constructed Genome for Allohexaploid Invasive Eurasian Watermilfoil ( *Myriophyllum spicatum* ) Reveals a Non‐Target Site QTL Associated With Fluridone Resistance

**DOI:** 10.1111/eva.70193

**Published:** 2026-01-09

**Authors:** Del Hannay, Gregory M. Chorak, Alex Harkess, Josh Clevenger, Josh T. Cuperus, Haley Hale, Laramie Aközbek, Zachary Meharg, Sarah B. Carey, Zachary Myers, Christine Queitsch, Arianna Stamatoyannopoulos, Ryan A. Thum

**Affiliations:** ^1^ Montana State University Bozeman Montana USA; ^2^ HudsonAlpha Institute for Biotechnology Huntsville Alabama USA; ^3^ University of Washington Seattle Washington USA; ^4^ Auburn University Auburn Alabama USA; ^5^ Dartmouth College Hanover New Hampshire USA

**Keywords:** aquatic plant management, contemporary evolution, genome assembly, herbicide resistance, invasive species, low coverage whole‐genome sequencing

## Abstract

Herbicides are a valuable tool in agricultural ecosystems to manage nuisance species. Due to the reliance on herbicides for weed control, herbicide resistance is a growing concern. Herbicides are also used extensively in aquatic and natural systems, but the genetics and evolutionary dynamics of resistance are not as frequently incorporated into management plans in these systems. In Eurasian watermilfoil, a widespread and heavily managed invasive aquatic weed in the United States, clonal lineages have been characterized as resistant to fluridone, a commonly used phytoene desaturase (PDS)‐inhibitor herbicide. In order to locate genomic loci associated with herbicide resistance, we created an F2 mapping population segregating for fluridone resistance. Using this population, we examined the *pds* gene for amino acid alterations in resistant individuals and performed bulk segregant analysis between the highly resistant and susceptible F2 individuals. Additionally, we compared *pds* gene expression between resistant and susceptible strains in control and treated environments using RT‐qPCR. We found no evidence of amino acid alterations to the *pds* gene in fluridone resistant individuals or increased *pds* expression in the resistant strain, either in the presence or absence of fluridone. Our QTL mapping identified a putative QTL on chromosome seven, while the gene encoding fluridone's target molecule, phytoene desaturase (PDS) is located on chromosomes 10–12. Our results indicate that fluridone resistance in the Eurasian watermilfoil strain isolated from Lake Lansing, MI, is due to at least one non‐target site mechanism. Characterizing mechanisms of herbicide resistance within invasive plants enables effective and thoughtful herbicide usage, as well as the development of diagnostic biomarkers for resistance in unknown populations.

## 1 Introduction

Since their introduction, herbicides have been a revolutionary and effective tool in global weed management, but their efficacy has been threatened in recent decades due to the extreme rise in herbicide resistance cases (Heap 2014). Herbicide resistance is a fascinating example of rapid contemporary evolution under strong human‐mediated selection pressures that can inform our understanding of evolutionary theory and mechanisms. In addition to how herbicide resistance can provide valuable insight for basic science, it also has major practical applications. The spread and impact of herbicide resistant weeds has and continues to threaten global food security and the mitigation of invasive species in natural ecosystems. In tandem, the drastic slowdown in the discovery and commercialization of new modes of action further exacerbates the difficulty in managing resistant weeds. Herbicide resistance is a critical and valuable research focus because it directly informs pest management and advances our understanding of evolutionary processes in a contemporary context.

The genetic architecture of the resistance (e.g., the number of genes involved, the distribution of their effects, and their interactions with each other and the environment) has large impacts on the spread, repeatability, and potential for resistance to arise (Hawkins et al. 2019; Jasieniuk et al. 2017; Powles and Yu 2010). The genetic architecture of resistance also has practical implications for management to respond to or mitigate the effects of resistance. For example, a major tool to combat failed herbicide treatments is the use of pre‐treatment screens for known resistance mutations (e.g., enhanced metabolic biomarkers in black‐grass, see Lowe et al. 2024; amino acid substitutions in crabgrass, see Basak et al. 2019). Therefore, characterizing the genetic mechanism(s) underlying resistance as it arises is a step towards developing management tools that integrate evolutionary frameworks and has the potential to improve management by predicting a population's response prior to herbicide treatment.

A common mechanism for herbicides is the inhibition or disruption of an enzyme or process critical for plant survival. The pathway that the herbicide disrupts is known as its mode of action (MOA) and the specific molecule it interacts with is known as the target site. There are two broad mechanisms of herbicide resistance at the molecular level: target‐site resistance (TSR) and non‐target site resistance (NTSR) (Gaines et al. 2020). TSR is the result of either structural changes in the herbicide binding site of the target molecule or over‐expression of the target gene (Delye et al. 2013; Jugulam and Shyam 2019). Because there is typically only one target site, TSR is a primarily monogenic trait. However, for herbicides with multiple target sites, such as very long chain fatty acid inhibitors, TSR is highly unlikely because it would require alterations to many genes. Thus, the MOA of the herbicide also plays a role in the potential for different resistance mechanisms. Conversely, NTSR encompasses a much broader range of how the plant copes with the herbicide, such as altered translocation, vacuolar sequestration, reduced absorption, or enhanced metabolism of the herbicide (Jugulam and Shyam 2019; Gaines et al. 2020). In NTSR cases, both polygenic and monogenic resistance traits are common, even in the same weed systems (Huffman et al. 2015). Multiple resistance mechanisms may also interact in complementary or antagonistic ways, potentially increasing the strength of resistance or cross resistance. Herbicides that inhibit the enzyme phytoene desaturase (PDS) have been effectively used to control nuisance plants and weeds for decades (Bartels and Watson 1978). PDS is a critical enzyme within the carotenoid biosynthesis pathway in photosynthetic organisms. Carotenoids protect plant cells from light damage; without these protective compounds, prolonged chloroplast damage will lead to plant bleaching and death. Although effective, resistance to PDS‐inhibitors (e.g., fluridone, norflurazon, etc.) has been observed in a few plant species (Dayan et al. 2014; Chorak and Thum 2020; Lu et al. 2020).

In two of the species, oriental mustard ( *Sisymbrium orientale* L.) and the invasive aquatic plant hydrilla ( *Hydrilla verticillata* L.f. Royle), resistant biotypes exhibit target‐site resistance through known structural mutations in the *pds* gene (Dang et al. 2019; Michel et al. 2004). In wild radish ( *Raphanus raphanistrum* L.) populations resistant to two PDS‐inhibitors, Lu et al. (2020) demonstrated that application of the cytochrome P450‐inhibitor malathion reversed resistance. These findings suggest some level of resistance is due to enhanced metabolism, a form of non‐target site resistance. In the remaining species, resistance mechanisms are either unknown or thought to involve NTSR, due to resistant biotypes being able to survive herbicides with differing MOAs (Dayan et al. 2014). No studies have yet identified non‐target site genetic associations with PDS‐inhibitor resistance.

Eurasian watermilfoil ( *Myriophyllum spicatum* L. sensu lato) is an aquatic allohexaploid macrophyte (Lü et al. 2017) of high management concern in North America. Across the United States, the PDS‐inhibitor fluridone has been used extensively to control Eurasian watermilfoil since 1987. The first documented case of fluridone resistance in watermilfoil was in 2012 (Berger et al. 2017; Thum et al. 2012). Chorak and Thum (2020) then documented that this resistant strain had spread between lakes in Michigan through clonal propagation and identified a second strain of watermilfoil resistant to fluridone isolated from Lake Lansing, Michigan. To date, the mechanism of fluridone resistance in these populations is unknown.

In this study, we investigate the genetic mechanism of fluridone resistance in one of two known fluridone‐resistant invasive Eurasian watermilfoil strains. In order to evaluate TSR mechanisms, we compared sequence variation and gene expression of the *pds* gene of resistant and susceptible genotypes. Additionally, we performed QTL bulk segregant analysis mapping to identify loci correlated with resistance using a full sibling segregating F2 population. All of this work was aided by the construction of a chromosome‐level annotated genome of the allohexaploid Eurasian watermilfoil species.

## 2 Methods

### 2.1 PacBio HiFi and Dovetail Omni‐C Sequencing

To assemble the genome of Eurasian watermilfoil, we collected fresh meristematic tissue from a culture in the Thum Lab greenhouse at Montana State University's Plant Growth Center (Bozeman, MT, USA). We chose the E_MISGP_734 genotype of Eurasian watermilfoil because it is widespread across the US (see Thum et al. 2020; Wolfe et al. 2025). Flash frozen tissue was then sent to HudsonAlpha Institute for Biotechnology (Huntsville, AL) for DNA extraction and sequencing.

We extracted high‐molecular‐weight (HMW) genomic DNA using a Takara NucleoBond HMW kit (San Jose, CA) using a modified version of the manufacturer's protocol. These modifications included: increasing lysis time to 1 h, using a Roto‐Therm (50°C at 20 rpm) during lysis, and omitting the air‐dry step after the ethanol wash. HMW genomic DNA was then sheared into 15 kilobase pair (kbp) fragments, and the HiFi library was prepared using SMRTbell Express Template Prep Kit 2.0 and the DNA/Polymerase Binding Kit 2.0 (Pacific Biosciences) according to the manufacturer's protocol. We size‐selected the sequencing library using Sage Blue Pippin (Sage Sciences) to select fragment sizes of > 10 kbp to ensure the removal of smaller fragments and adapter dimers. We then sequenced the library on a PacBio Sequel II instrument in CCS/HiFi mode with two SMRT cells for 2 h of pre‐extension and 30‐h movie times. We assessed read length distribution and quality of all HiFi reads using Pauvre (Shultz et al. 2019). To scaffold the genome using chromatin conformation sequencing, we harvested 1 g of flash‐frozen young leaf material. The sequencing library was prepared using the Dovetail Genomics Omni‐C kit and was sequenced on an Illumina NovaSeq 6000 with PE150 reads. We then used a subset of the 1 million read pairs as input for Phase Genomics hic‐qc (v 1.0) to validate the overall quality of the library.

### 2.2 Assembly and Scaffolding

Based on flow cytometry, the expected genome size is ~780 Mb (Hidalgo et al. 2015). We counted k‐mers in the Illumina Omni‐C reads using Genomescope 2.0 (Ranallo‐Benavidez et al. 2020). We found a similar pattern in the k‐mer spectra plots as the example allohexaploid plots in Genomescope 2.0 (Figure S1), supporting the hypothesis that Eurasian watermilfoil is an allohexaploid. We assembled the HiFi reads into contigs using hifiasm v0.16.0 (Cheng et al. 2021, 2022), with the Hi‐C integration mode, which incorporates Dovetail Omni‐C reads for phasing. We then scaffolded the assembly into chromosomes using the Yet Another Hi‐C Scaffolding (YAHS) tool version 1.1. First, Omni‐C reads were mapped using bwa mem version 0.7.17 with parameters –5SP and samtools version 1.10 with parameters view –S –h –b –F 2316. Next, we scaffolded the assembly using YAHS with default parameters. We edited contact maps manually with Juicebox Assembly Tools (JBAT) version 1.9.9 (Durand et al. 2016) to produce the expected 21 chromosomes and manually rearranged chromosomes in JBAT so that homoeologous chromosomes were next to each other. Homoeologous chromosomes share more contact with each other than non‐homoeologous chromosomes (See Figure 2). We ensured the proper orientation of the telomeres by searching for telomeric repeat (AAATCCC)_*n*_ and plotted their location, along with contig breaks using GENESPACE v1.3.1 (Lovell et al. 2022). We checked for contamination in contigs using FCS‐GX version 0.4.0 using tax‐id 208873 to ensure contaminant contigs did not end up in the 21 chromosomes and remove unincorporated contaminant contigs. We then assessed genome quality and completeness using benchmarking universal single‐copy gene orthologs (BUSCO v5.4.4; Manni et al. 2021) with the “eudicots_odb10” database.

### 2.3 Transcriptome Sequencing

To facilitate gene annotation, RNA was isolated from five meristems of the same genotype sequenced for genome assembly (E_MISGP_734). We isolated total RNA using a Qiagen RNeasy Extraction kit (Valencia, CA, USA). We checked RNA quantity on a Qubit 4 fluorometer (ThermoFisher Waltham, MA, USA) and submitted at least 1 μg of RNA to the University of Minnesota Genomics Core (UMGC; Minneapolis, MN, USA) for Illumina TruSeq Stranded mRNA Library Prep (San Diego, CA, USA), following the manufacturer's instructions. After library prep, UMGC paired‐end sequenced each sample on an Illumina NovaSeq 6000 (San Diego, CA, USA) using 150 bp inserts.

### 2.4 Repeat Analysis and Gene Annotation

We first annotated repetitive elements using EDTA v2.0.0 (Ou et al. 2019) with flags “–genome, –anno 1, –sensitive 1.” We then used the repeat masked genome output from EDTA as input into the gene annotation software BRAKER3 (Gabriel et al. 2024), along with the raw RNA‐seq reads from above and the OrthoDB v.11 (Kuznetsov et al. 2023) eukaryota protein database as inputs. Next, we used the BRAKER3 coding sequence “.fasta” output as input into the Blast2GO pipeline (Gotz et al. 2008) in BioBam‐Omics Box software (Version 3.0.30 OmicsBox ‐ Bioinformatics Made Easy 2019). We first used DIAMOND (Buchfink et al. 2021) to do a basic local alignment (BLAST) of BRAKER3 identified coding sequences against the Swissprot (2024‐11‐20) database using the “more sensitive” setting. Next, we used InterProScan 5.72‐103.0 (Jones et al. 2014) to search for protein families, domains, sites, and repeats of the entire InterProScan member database set. Finally, we used the default settings of Blast2GO (Gotz et al. 2008) to perform gene ontology (GO) mapping and annotation.

### 2.5 Mapping Population Construction

To build a mapping population that segregates for fluridone resistance, we crossed a known fluridone resistant genotype isolated from Lake Lansing, Michigan (MG‐377 from Chorak and Thum 2020; E_MISGP_380 in Wolfe et al. 2025), with a known fluridone susceptible genotype isolated from Base Line Lake, Michigan (MG‐1282 from Chorak and Thum 2020; H_MYR_10199 in Wolfe et al. 2025). Henceforth, these genotypes will be referred to by the names documented in Wolfe et al. (2025) and at https://thumlab‐msu‐watermilfoilapp.shinyapps.io/milfoil_app/. To cross these two genotypes, we used a similar method to those found in Thum and McNair (2018). Briefly, we cultured each genotype by planting several meristems in soil (peat‐topsoil‐sand 1‐1‐1) in separate 208‐L tanks filled with Smart and Barko (1985) buffered water. Culture tanks were kept in a natural light greenhouse at Montana State University's Plant Growth Center (Bozeman, MT) with additional supplemental lighting to maintain 16 h light: 8 h dark days. Watermilfoil produces bisexual flower stalks, with male (stamen) flowers on the top and female (pistil) flowers on the bottom. The flowers develop from the bottom of the stalk to the top of the stalk, so female flowers open first, and males open within 1–3 days later. To ensure that seeds produced were from the cross between the resistant and susceptible genotypes (and not self‐pollinated), we set one culture as the female tank that would receive pollen and removed the male part of the flower stalk before it opened. The other culture was allowed to mature to the point of open male flowers and then was picked and used to brush the pollen on the female flowers of the receiving culture. We collected fruits from the female culture tank and placed them into Ziploc bags with culture water to ripen. Once fruits were ripened, we removed the endocarp around the seed and placed clean seeds in deionized water and stored in the fridge until germination. This whole process was repeated to perform crosses in the opposite direction, so the initial progeny were constructed from crosses in both directions.

To germinate the F1 progeny, we placed the seeds in an open container filled with deionized water. Once plants had developed to the two‐leaf stage, they were planted into Cone‐tainers (Stuewe and Sons Inc., Tangent, OR) with soil (peat:topsoil:sand 1:1:1) and pressed into the sand‐topped soil bottom of a 378.5‐L tank filled with Smart and Barko (1985) buffered water. Progeny were cultured in a common garden in a greenhouse with supplemental lighting at Montana State University's Plant Growth Center (Bozeman, MT), and each individual was labeled and monitored. We then crossed F1 individuals randomly with each other in order to create an E_MISGP_380 × H_MYR_10199 F2 population. The F2 population was germinated and cultured in the same procedure as the F1 progeny above.

### 2.6 Segregation Experiment and Genotyping

We conducted a fluridone selection experiment with the F2 progeny at 6 μg L^−1^ fluridone using methods similar to those found in Chorak and Thum (2020). Briefly, a 10‐cm meristem from each of the 308 progeny was replanted into new Cone‐tainers (Stuewe and Sons Inc., Tangent, OR) with soil (peat‐topsoil‐sand 1‐1‐1) and pressed into the soil‐lined bottom of a 378.5‐L tank filled with Smart and Barko (1985) buffered water. Individuals were equally divided into five steel cattle tanks. After the two‐week establishment period, all five tanks were treated with 6 μg L^−1^ fluridone. Meristematic tissue samples were taken from the F2 individuals prior to treatment for later sequencing. After 60 days in fluridone treatment, we harvested plants and dried them in a drying room at Montana State University's Plant Growth Center (Bozeman, MT, USA). We then measured dry biomass for each plant.

**FIGURE 1:**
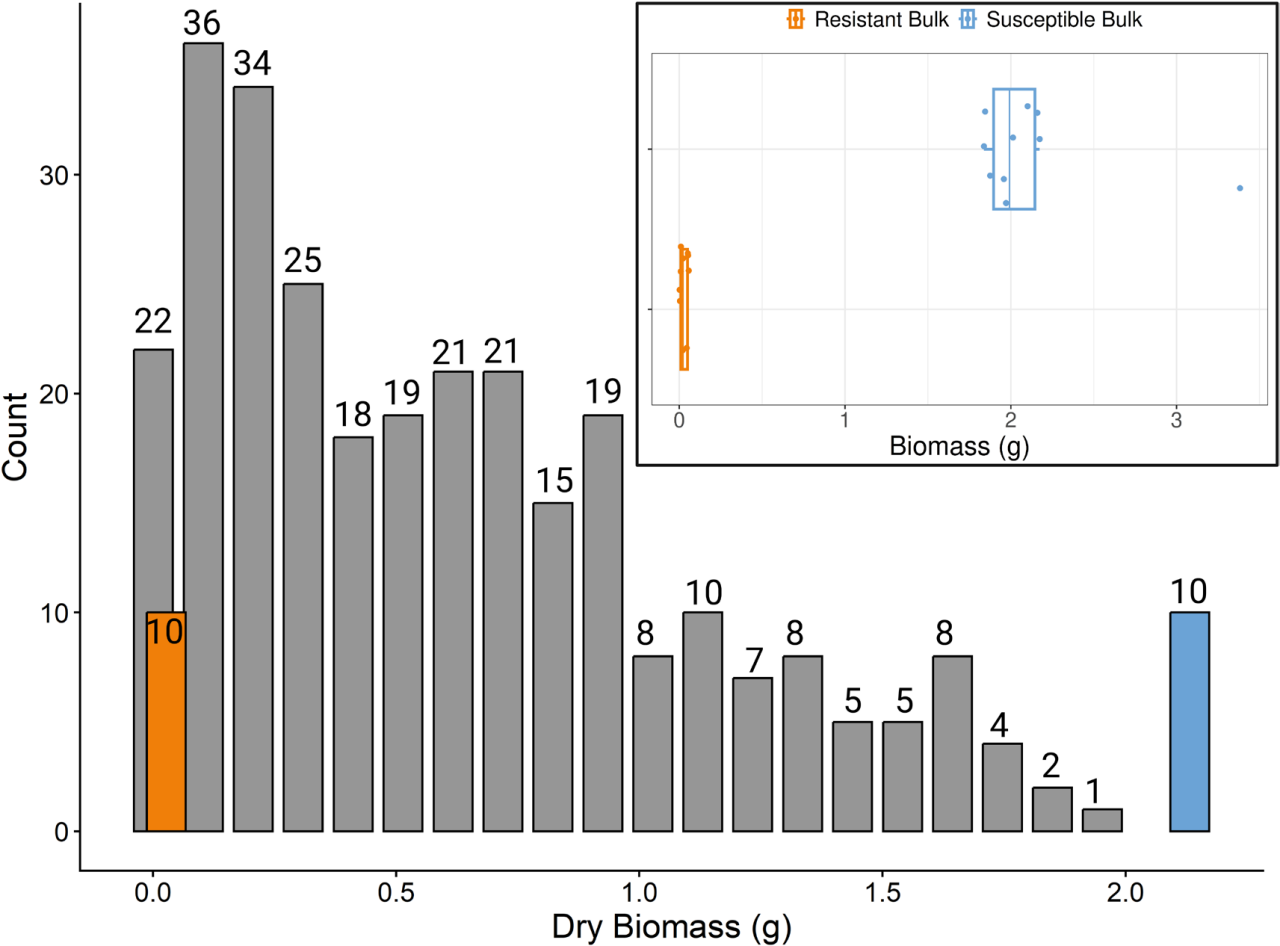
Distribution of F2 mapping population's dry biomass measurements after fluridone treatment (*n* = 308). Bars are centered around the mean for each bin. Inset boxplot shows the dry biomass measurements for the ten highly susceptible (orange) and ten highly resistant (blue) individuals sequenced for QTL analysis. Numbers above bars represent counts.

From biomass measurements of the population, we saw clear evidence for variation in the degree of resistance versus susceptibility and we identified 10 highly susceptible and 10 highly resistant individuals to 6 μg L^−1^ of fluridone (Figure 1). The mean dry biomass of the susceptible bulk was 0.028 g and the mean dry biomass of the resistant bulk was 2.132 g. We used the Qiagen DNeasy Plant Mini DNA extraction kits (Valencia, CA, USA) to extract whole DNA from frozen tissue samples of the F2 bulk individuals as well as one sample of each parental genotype. DNA was then sent to the HudsonAlpha Institute for Biotechnology for paired‐end Illumina short‐read low‐coverage, whole‐genome resequencing on a NovaSeq 6000 system.

We processed the low coverage whole‐genome resequencing (lcWGS) data from the highly resistant and susceptible F2 individuals and their parents via the pipeline described below. Adapters and low‐quality reads (PHRED < 20) were trimmed using the bbduk function from the BBMap suite (Bushnell 2025). Paired reads were also trimmed to the same length. Hisat2 was used to index our reference Eurasian watermilfoil genome and then align forward and reverse reads (Kim et al. 2019). Alignment map files were sorted and indexed using SAMtools (Danecek et al. 2021). Next, the susceptible and resistant individual samples were merged into bulk alignment map files. We then performed variant calling on the bulks and parents using BCFtools mpileup, call, annotate, and view functions (Danecek et al. 2021). Variants with a mapping quality lower than 30, a read depth less than 10, a minimum base quality lower than 20, and a strong strand bias (Fisher's score greater than 60 or more than 80% of alternate allele reads from a single strand) were excluded at this step. Setting a high mapping quality ceiling ensured multi‐mapped reads would not be included in the final dataset, which is often a challenge with polyploid species.

### 2.7 Sequence Variation of *pds* Gene

To evaluate whether fluridone resistance in the E_MISGP_380 lineage is due to structural mutation(s) in the PDS molecule, we compared mutations in the *pds* gene. Because Eurasian watermilfoil is an allohexaploid, we examined the reads that aligned to the three copies of *pds* in the reference haploid genome. Additionally, the genotype used to create the reference genome is susceptible to fluridone, so we would expect resistant genotypes to have an alternate allele at sites associated with resistance. We used the lcWGS data to compare variants between resistant and susceptible bulks and between resistant and susceptible parents within the pds genes on chromosomes 10 (35,658,175–35,664,873 bp), 11 (2,953,758–2,960,399 bp), and 12 (35,497,980–35,504,980 bp). We then used our gene annotation to determine whether variants were located in intronic or exonic sequences and whether variants would result in amino acid changes in the *pds* gene.

### 2.8 Expression of *pds* Gene

To determine if fluridone resistance is due to differences in PDS molecule abundance, we measured *pds* gene expression of the fluridone resistant E_MIGP_380 and fluridone susceptible H_MISGP_457 strains using quantitative reverse transcription polymerase chain reaction (RT‐qPCR). Unfortunately, the mapping population's susceptible parent culture was not growing well and could not be used as a control in this experiment. Therefore, we used a susceptible reference with a similar fluridone response as the susceptible parent. One experiment consisted of three identical 273‐L tanks, grown in a controlled growth chamber at Montana State University's Plant Growth Center. The second experiment consisted of three identical 189‐L tanks grown in a greenhouse with both natural and artificial light. These experiments were performed in different conditions to reduce the effect of environmental factors on gene expression. Common garden tanks each contained six replicates of the two strains (E_MISGP_380 and H_MISGP_457), for a total of 12 individuals per tank.

Vegetative propagules for the experiment were taken from the individual genotype cultures grown in Montana State University's Plant Growth Center. For the first experiment, meristematic clippings of uniform 10 cm lengths were taken from individual cultures and planted in Cone‐tainers (Stuewe and Sons Inc., Tangent, OR, USA) with soil (1:1:1 peat:topsoil:sand) topped with pure sand. Cone‐tainers were arranged randomly into the sand‐topped soil of the tanks. In the second experiment, genotypes were planted into 19 L buckets with sand‐topped soil within the barrels and placed into 189 L barrels containing Smart and Barko's general purpose culture solution (Smart and Barko 1985). We aerated the tanks using an aquarium air bubbler. In the second experiment, tank heat was maintained between 20°C and 22°C via aquarium heaters. Heaters were not needed in the heat‐controlled growth chamber used in experiment one. To prevent the effect of previous growing conditions contributing to experimental results, individuals were allowed to grow for 3 weeks until plants had well‐established roots and new shoot growth. After this establishment period, tissue samples were randomly taken from half of the individuals of each genotype in each tank. We then treated each tank with 6 μg L^−1^ of fluridone (Sonar Genesis, SePRO, Carmel, IN, USA) and monitored plants for the physical symptoms of fluridone. At the onset of the first bleaching symptoms in the stems and leaf nodes of any individual, all remaining individuals were sampled and flash‐frozen until further use.

We extracted RNA from frozen tissue samples using the Qiagen RNeasy Plant Mini extraction kit following manufacturer protocol. To standardize the total RNA used in cDNA synthesis, we quantified RNA concentration using a Qubit RNA Broad Range (BR) Assay Kit and then took 500 ng of total RNA from each sample into the next step. RNA samples were cleaned using the Invitrogen TURBO DNA‐free Kit, and cDNA was synthesized using the Invitrogen SuperScript IV First‐Strand Synthesis System and ezDNase Enzyme Kit.

All qPCRs were performed on a Bio‐Rad CFX384 machine. To validate and compare *pds* expression, a gene of consistent expression (reference gene) was used to standardize *pds* expression in all samples, following the suggested procedure of Xu et al. (2012). qPCR primers for both *pds* and reference genes were designed using the Integrated DNA Technologies (IDT) PrimerQuest Tool. We selected primers with a melting temperature of 72°C and a GC content between 45% and 55% to amplify approximately 100 bp. We tested four *pds* primers and four reference gene primers. The reference genes tested were *otu1* (ubiquitin thioesterase), *afl7* (histone binding zinc finger), and *rad23b* (ubiquitin binding). We tested two primer sets from different sections of *otu1*. We resuspended primers at 10× concentration, and qPCR was performed in 20 μL reactions consisting of 2 μL of cDNA, 6 μL RNase‐free water, 10 μL 2× iTaq Universal SYBR Green Supermix, and 2 μL of 10× PrimeTime Assay primer. The PCR cycle procedure consisted of 25 s at 95°C, 40 rounds of 5 s at 95°C, and 25 s at 56.3°C. The melting curve was generated from 65°C to 95°C at 5 s increments.

We tested the specificity of each primer by the presence of a singular ~100‐bp band on 1% agarose gel electrophoresis, a singular melting curve peak during qPCR, and a qPCR efficiency value around 100% (Bustin and Huggett 2017; Derveaux et al. 2010). To create standard qPCR curves, pooled cDNA samples were diluted over 5 log_10_ of magnitude for all eight primers. Through these parameters, one reference primer from the *otu1* gene and one *pds* primer was selected for forward analyses of the common garden samples.

All qPCR samples were performed in technical triplicate. Each qPCR plate contained pooled cDNA samples diluted over 5 log_10_ of magnitude for standard curves, as well as a PCR negative control.

We calculated the relative expression of *pds* using the 2^−ΔΔCq^ method (Livak and Schmittgen 2001), in which the delta quantification cycle (*ΔC*_*q*_) was calculated by subtracting the *otu1 C*_*q*_ value from the *pds C*_*q*_ value for each biological sample (technical replicates were averaged). Then, control *ΔC*_*q*_ values for each tank and genotype were averaged, and this average *ΔC*_*q*_ was subtracted from the treated *ΔC*_*q*_ values for each tank and genotype. We then calculated the fold‐change with the equation 2^−Δ*ΔCq*^.

We performed all statistical analyses in RStudio V2023.06.0+Build 421 (R Core Team 2024; Posit team 2025). To assess genotype, treatment, or their interaction effect on fold‐change, we created a linear mixed‐model of *pds* fold change using the R packages *lme4* (Douglas Bater and Ben Bolker 2015) and lmerTest (Kuznetsova et al. 2017) with tank and experiment block as a random effect (Fold Change ~ Genotype × Treatment + [1|Tank] + [1|Experiment]). We then performed an analysis of variance (ANOVA) type III using Satterthwaite's method.

### 2.9 QTL Analysis

We used the qtlplot function in the program *QTL‐seq* to map SNP differences between the resistant and susceptible bulks (Takagi et al. 2013; Sugihara et al. 2020). Because our reference genome was not created using a parental genotype from this cross, the variant dataset was further filtered for sites where the susceptible parent was homozygous for the reference allele. Briefly, QTL‐seq compares the proportion of reference and alternate alleles at each polymorphic site between the two phenotypic extreme bulks (i.e., a ΔSNP index). An average ΔSNP index was calculated for each 2‐Mbp window, with a step size of 100 kbp between each window. The significance threshold for a putative QTL was created via 5000 permutations of expected ΔSNP indices under the null hypothesis (no QTL present). SNPs with a read depth lower than 25 reads or higher than 250 (to avoid incorrectly inflated reads) were excluded from the analysis.

## 3 Results

### 3.1 A Chromosome‐Scale Assembly

**FIGURE 2:**
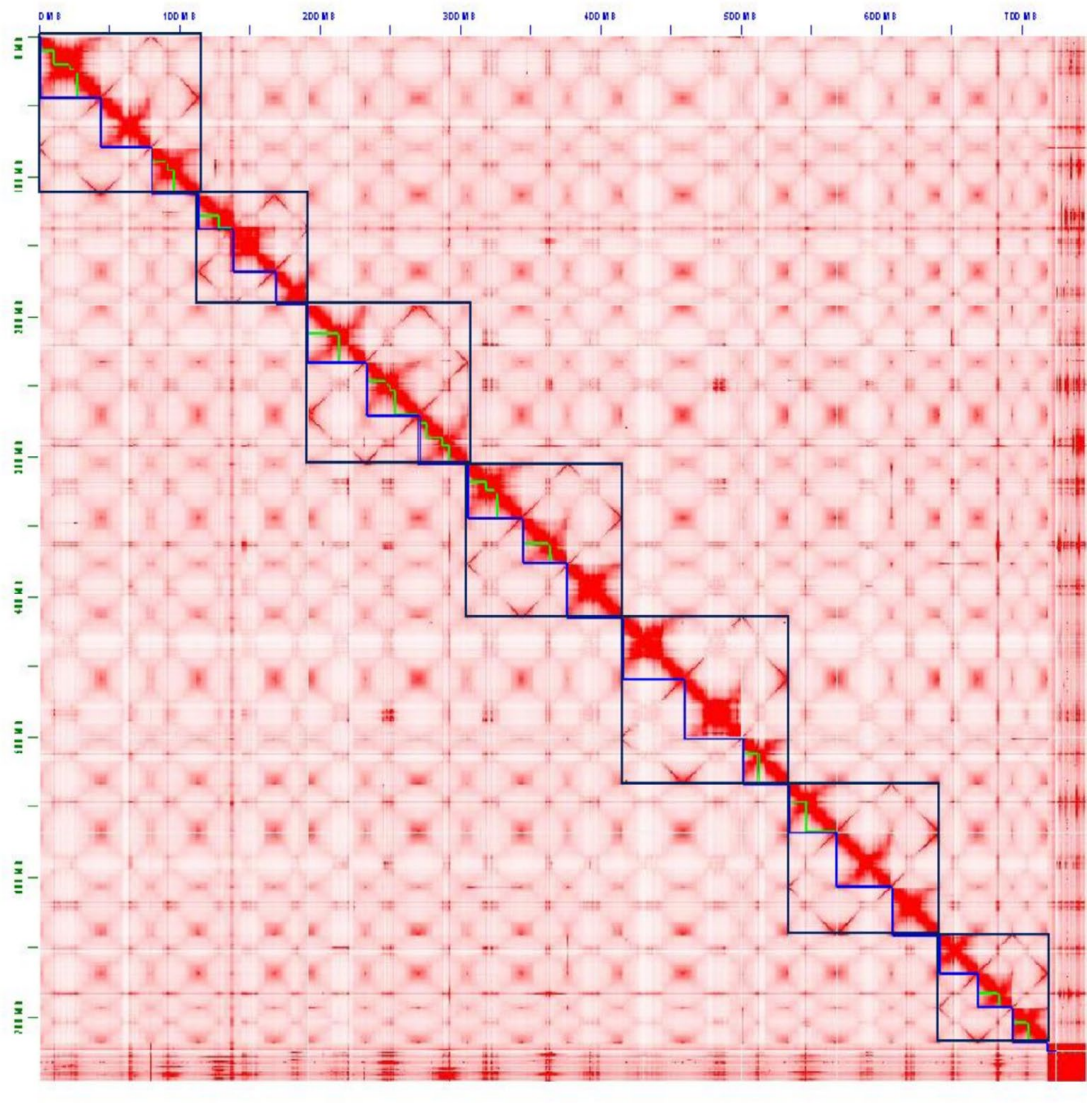
A Hi‐C interaction plot of each of the sequences used to generate the *Myriophyllum spicatum* genome assembly. Each of the blue half boxes represents the 21 chromosome scaffolds. The larger dark blue boxes group the chromosome scaffolds that are homoeologous.

In total, we generated nearly 100× coverage of PacBio HiFi reads in three runs and > 200× coverage of Dovetail Omni‐C reads over two runs. This included ~4.37 million HiFi reads with an average length of 17,820 base pairs (bp) and ∼96% of reads ≥ 10,000 bp (Figure S2). The final assembly is ~743 megabase pairs (Mbp) in length, contained in 453 contigs and 276 scaffolds with a scaffold N50 of 34.7 Mbp. All 21 primary chromosome scaffolds are larger than 10 Mbp, and the majority of the remaining scaffolds are less than 100 kilobases (Table S1). We did not need to do any manual joins or breaks after JBAT assembled contigs into scaffolds (Figure 2). The 21 chromosomes contain ~97% of the assembled sequence, and 98.8% of the benchmarking universal single‐copy orthologs (BUSCOs) were complete in our assembly (Figure S3). No contamination was found in the 21 primary scaffolds using FCS‐GX, and contamination in excess scaffolds was excluded from the final assembly (Table S2). Of the 21 chromosomes, 12 contained significant telomeric sequence on both terminal ends, and the remaining nine contained telomeres on at least one end (Figure S4). The genome was published in the National Center for Biotechnology Information (BioSample accession SAMN52826724).

### 3.2 Genome Annotation

The yield of Illumina transcriptome sequencing from meristematic tissue was ~401 million reads, resulting in ~60 gigabase pairs (Gbp). Repetitive elements made up 39.9% of the assembly, mostly consisting of long terminal repeats (LTR; 17.82%). A total of 100,854 genes were annotated using BRAKER3 and of those, 61,340 had predicted functions for at least one GO term using the Blast2Go pipeline accessed within OmicsBox. Our genome annotation returned 88.7% BUSCOs (Figure S2).

### 3.3 Sequence Variation of *pds* Gene

To determine if fluridone resistance in a strain of Eurasian watermilfoil is due to structural alterations of the PDS molecule, we compared mutations present in the *pds* gene between fluridone resistant and susceptible full‐sibling individuals, as well as the parent genotypes. We would expect mutations conferring fluridone resistance in *pds* to be found only in the resistant parent and resistant F2 bulk and not in the susceptible parent or bulk.

Because Eurasian watermilfoil is an allohexaploid, the *pds* gene is found on three chromosomes in our genome assembly (chromosomes 10–12). Overall, there were no mutations exclusive to resistant genotypes (Table 1). More often, mutant alleles at variant sites were found in the susceptible parent. One missense mutation, at chromosome 10 position 35,664,868 bp, encoded a change from serine to alanine. The resistant parent was reference homozygous (T/T) and the susceptible parent heterozygous (T/G) at this site. The mutant allele was found in 17.4% of the susceptible and 3.7% of the resistant F2 bulk reads. Because the mutant allele was found in the susceptible parent and a small proportion of both F2 bulks, we concluded this mutation to not be associated with the resistance trait. A second missense mutation found on chromosome 11 position 2,953,806 bp encoded a change from glutamic acid to aspartic acid. Again, the resistant parent was reference homozygous (A/A) and the susceptible parent was heterozygous (A/T). The mutant allele was found in 5.6% of the susceptible and 21.4% of the resistant F2 bulk reads. Because this mutation was shared between the susceptible parent and both bulks, this mutation is also not associated with resistance. All other mutations found in the chromosome 10 and 11 copies of the *pds* gene were either silent mutations or found within intronic regions and therefore would not have an impact on the structure of the PDS enzyme.

**TABLE 1:** Variant sites and their predicted effects in the *pds* gene between the E_MISGP_380 x H_MYR_10199 F2 bulks and the parental genotypes. Variant effects could not be determined for chromosome 12 due to premature stop codons throughout that *pds* copy.

Chromosome	DNA position	Reference allele	Mutant allele	Resistant parent genotype	Susceptible parent genotype	Proportion of mutant allele reads in the susceptible bulk	Proportion of mutant allele reads in the resistant bulk	Effect
10	35,658,186	C	T	C/C	C/T	0.125	0.089	Silent
10	35,662,307	G	C	G/G	G/C	0.250	0.000	None
10	35,662,662	G	T	G/G	G/T	0.125	0.000	None
10	35,663,623	T	A	T/T	T/A	0.389	0.071	None
10	35,663,841	G	T	G/G	G/T	0.333	0.059	None
10	35,664,868	T	G	T/T	T/G	0.056	0.214	Missense
11	2,953,806	A	T	A/A	A/T	0.174	0.037	Missense
11	2,953,814	C	T	C/C	C/T	0.208	0.061	Silent
11	2,954,901	A	G	A/A	A/G	0.500	0.037	None
11	2,955,532	A	G	A/A	A/G	0.100	0.037	None
11	2,955,536	G	A	G/G	G/A	0.111	0.034	None
11	2,955,546	G	A	G/G	G/A	0.115	0.034	None
11	2,955,767	C	A	C/C	C/A	0.444	0.071	None
11	2,955,799	T	A	T/T	T/A	0.400	0.077	None
11	2,955,931	T	C	T/T	T/C	0.000	0.375	None
11	2,957,290	A	G	A/A	A/G	0.636	0.000	None
11	2,958,274	G	T	G/G	G/T	0.353	0.000	None
11	2,958,303	C	A	C/C	C/A	0.294	0.000	None
11	2,958,309	G	T	G/G	G/T	0.143	0.000	None
11	2,958,527	G	A	G/G	A/A	0.000	0.154	None
11	2,959,218	A	T	A/A	A/T	0.235	0.000	None
11	2,959,290	C	A	C/C	C/A	0.318	0.040	None
12	35,498,038	G	C	G/C	G/G	0.200	0.563	Unknown
12	35,498,182	T	G	G/G	T/G	0.900	0.762	Unknown
12	35,498,799	C	A	C/C	C/A	0.000	0.000	Unknown
12	35,498,909	C	T	C/C	C/T	0.750	0.000	Unknown
12	35,498,976	G	A	G/G	G/A	0.762	0.000	Unknown
12	35,499,049	A	G	A/A	A/G	0.348	0.000	Unknown
12	35,500,142	A	T	A/A	A/T	0.778	0.000	Unknown
12	35,500,670	A	G	A/A	A/G	0.185	0.250	Unknown
12	35,500,758	T	C	T/T	T/C	0.300	0.214	Unknown
12	35,500,899	T	G	T/G	T/T	0.188	0.364	Unknown
12	35,501,336	A	G	A/A	A/G	0.385	0.000	Unknown
12	35,501,484	C	A	C/C	C/A	0.500	0.059	Unknown
12	35,502,447	T	C	T/T	T/C	0.273	0.000	Unknown
12	35,503,417	C	T	C/C	C/T	0.000	0.000	Unknown
12	35,504,350	C	T	C/C	C/T	0.571	0.100	Unknown
12	35,504,521	T	C	T/T	T/C	0.700	0.000	Unknown
12	35,504,644	T	G	T/T	T/G	0.444	0.000	Unknown
12	35,504,732	T	A	T/T	T/A	0.400	0.000	Unknown
12	35,504,753	T	C	T/T	T/C	0.368	0.000	Unknown

The chromosome 12 copy of *pds* appeared to be non‐functional, with premature stop codons throughout the annotation. Therefore, exonic and intronic regions and reading frames could not be confidently annotated. Nevertheless, we still assessed mutations that mapped within the entire *pds* region on this chromosome (positions 35,497,980–35,504,980 bp). We would expect mutations conferring resistance to only be found in the resistant parent and resistant F2 bulk and not in the susceptible parent or bulk. In total, we found two sites where the resistant parent was heterozygous for the reference allele and a mutant allele. At both sites, the susceptible parent was reference homozygous. However, the F2 bulks, regardless of resistance trait, had both alleles. Because we would expect a causal mutant allele to be only associated with resistant individuals and the susceptible progeny have this allele, we do not believe these mutations are associated with resistance (Table 1).

**FIGURE 3:**
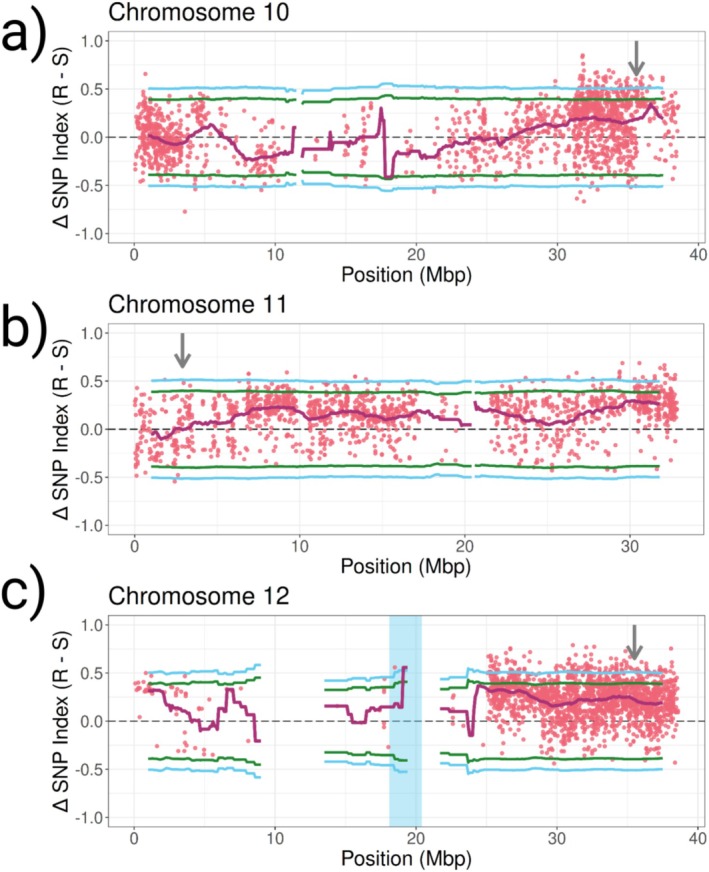
QTL mapping results for chromosomes 10–12 (a–c). ΔSNP‐index values (R—S) between resistant and susceptible F2 individuals are represented by pink dots. Magenta lines are mean calculated ΔSNP‐indices for 2 Mbp windows with a step size of 100 kbp. Blue and green lines represent the 99th and 95th confidence intervals for a null ΔSNP‐index distribution calculated from 5000 replicated permutation tests. Grey arrows point towards the approximate location of the target site gene *pds* on each chromosome.

In addition, we performed QTL‐seq analysis (see Section 3.5), which allowed us to search for selection signatures within or around the *pds* gene. The average delta SNP‐index of the 2Mbp regions encompassing each *pds* copy was 0.174, 0.002, and 0.225 for chromosomes 10, 11, and 12, respectively (Figure 3). We conclude from this that there is no evidence of an association between variants in these regions and fluridone resistance. These results, combined with the previous narrow examination of sequence variation in the bulks and parents, provide no evidence of a structural mutation in the *pds* gene impacting response to fluridone.

### 3.4 Expression of *pds* Gene

To determine whether over‐expression of the target molecule may cause fluridone resistance, we quantified the fold change of the *pds* gene using RT‐qPCR. We determined *pds* gene expression differences in the fluridone resistant parent used in the mapping population of this study and a fluridone susceptible reference strain in two common garden experiments. The *pds* gene expression was normalized using the *otu1* gene.

**FIGURE 4:**
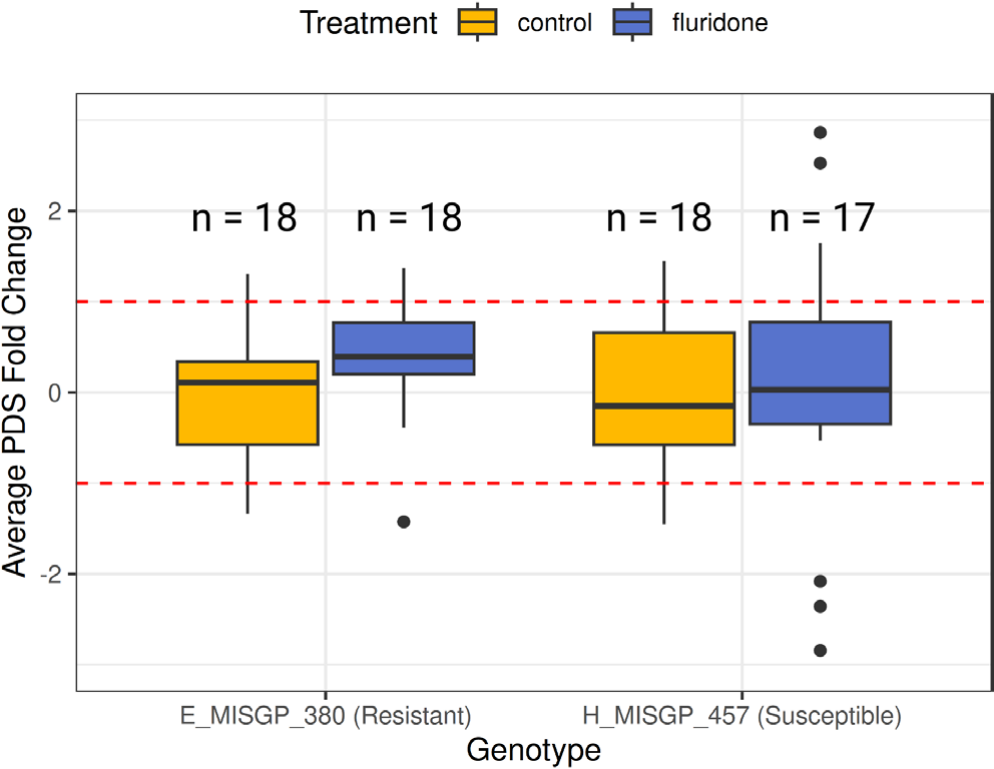
Comparison of *pds* log_2_ fold change between a fluridone resistant genotype (E_MISGP_380) and fluridone susceptible genotype (H_MISGP_457). The *otu1* gene was used as a reference gene. Colors of boxplots and points correspond to treatment levels: Plants with no fluridone exposure (control, yellow) and plants treated with 6 μg L^−1^ of fluridone (blue). Red dotted lines represent one PCR cycle on a log_2_ scale. Numbers above boxes represent sample sizes.

We found no evidence (p‐value 0.315) that the resistant strain's mean *pds* expression is different from the susceptible reference (Figure 4, Table S3). Additionally, we found no evidence (*p*‐value 0.831) of a fluridone treatment effect on *pds* fold change expression in either strain. These results indicate that an increase in *pds* expression, constitutive or facultative, is not responsible for fluridone resistance in the E_MISGP_380 genotype.

### 3.5 QTL Analysis

After determining there was no evidence for target site resistance, we aimed to identify genomic regions associated with fluridone resistance in an F2 population segregating for fluridone response using QTL‐seq. Ten highly resistant and ten highly susceptible individuals, determined via biomass after 9 weeks of 6 μg L^−1^ fluridone exposure, were selected for whole genome sequencing and aligned to the Eurasian watermilfoil reference genome along with the parental genotypes.

**FIGURE 5:**
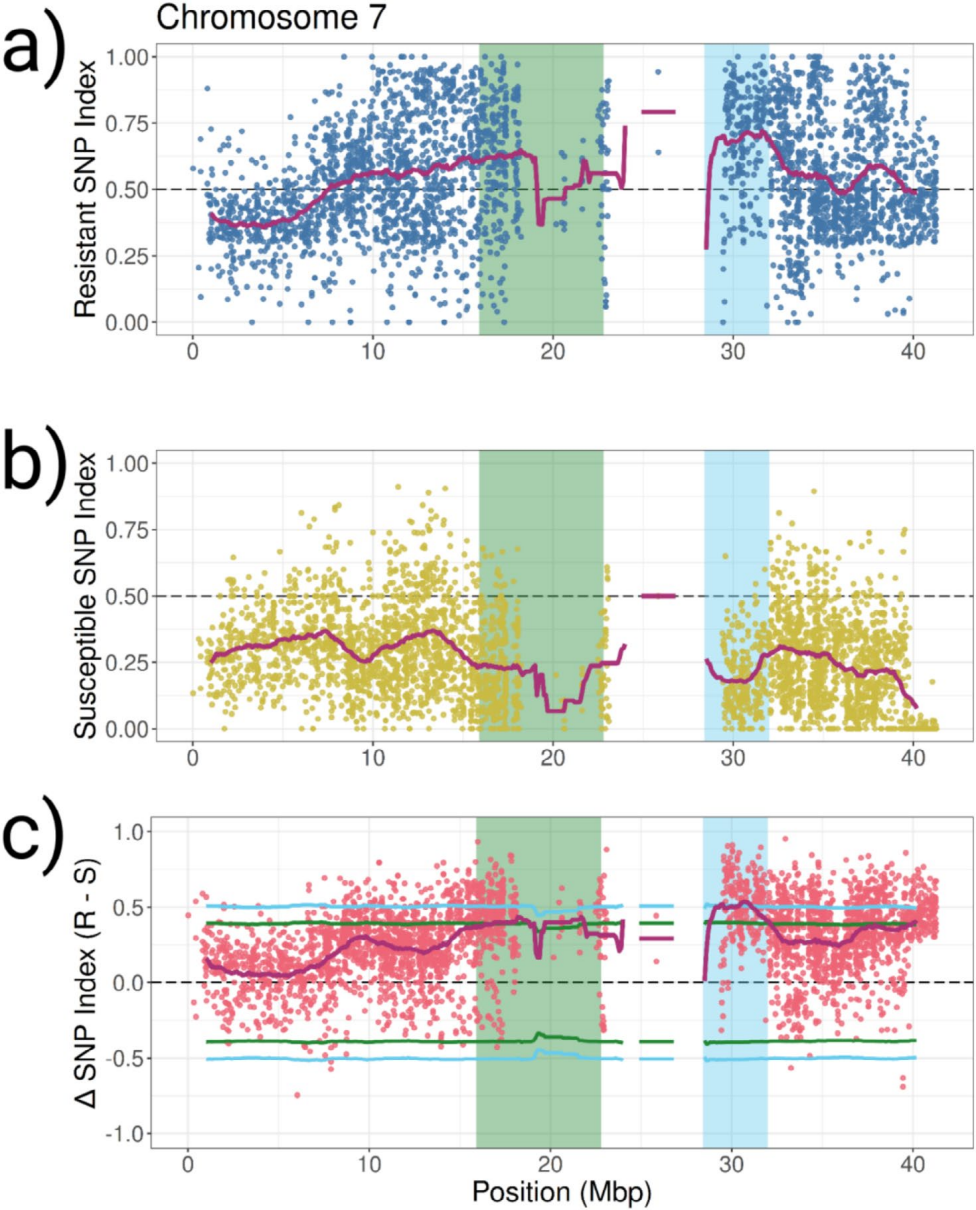
QTL mapping results for chromosome seven. (a) SNP indices (blue dots) of 10 resistant F2 individuals. (b) SNP indices (yellow dots) of 10 susceptible F2 individuals. (c) ΔSNP‐index values (R–S; pink dots) between resistant and susceptible F2 individuals. Magenta lines in all three panels are mean calculated SNP indices for a 2 Mbp window. Blue and green lines in panel C represent the 99th and 95th confidence intervals for a null ΔSNP‐index distribution. The regions above the 99th and 95th percentiles are highlighted in blue and green on all three panels, respectively.

**FIGURE 6:**
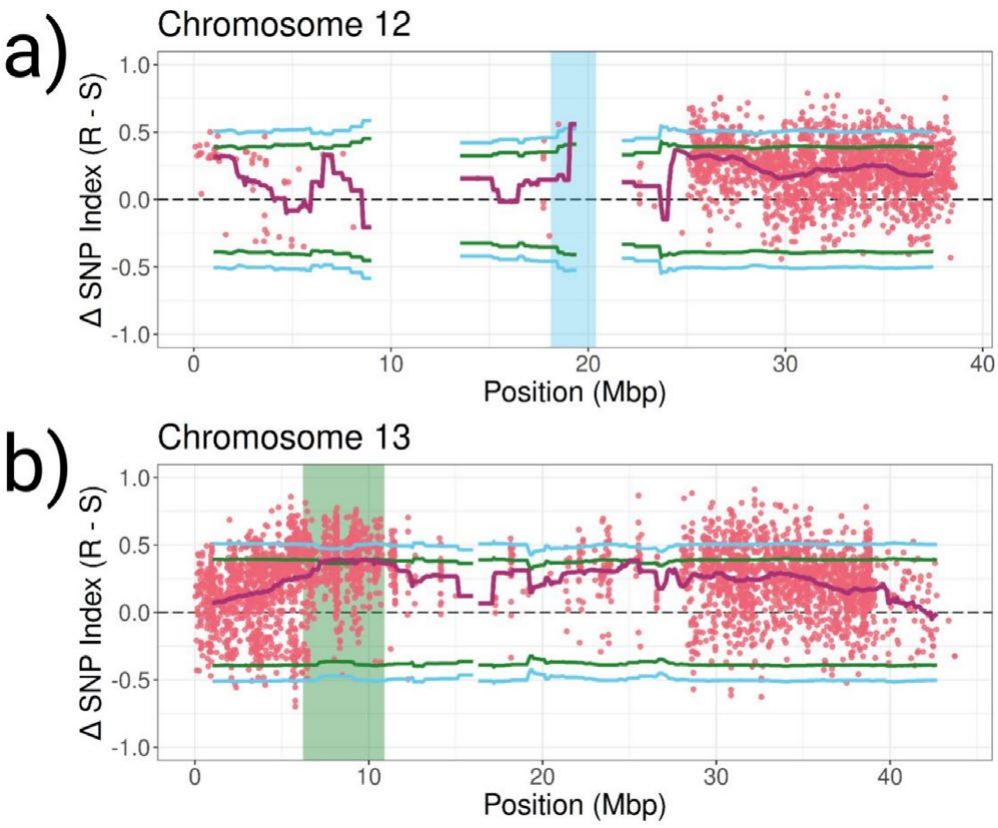
QTL mapping results for chromosomes 12 (a) and 13 (b). ΔSNP‐index values (R–S) between resistant and susceptible F2 individuals are represented by pink dots. Magenta lines are the mean calculated ΔSNP‐indices for 2 Mbp windows with a step size of 100 kbp. Blue and green lines represent the 99th and 95th confidence intervals for a null ΔSNP‐index distribution calculated from 5000 replicated permutation tests. The regions above the 99th and 95th percentile are highlighted in blue and green, respectively.

The proportion of each allele at a polymorphic site (SNP‐index) was then compared between the two bulks and a ΔSNP index is created (Resistant SNP‐index—Susceptible SNP‐index). A ΔSNP index of 1 therefore indicates every individual in the resistant bulk had the alternate allele and every individual in the susceptible bulk had the reference allele. An expected distribution of ΔSNP‐indices under the null hypothesis of no QTL present was calculated in order to identify regions outside these expectations. One region from 28.4 to 32.0 Mbp on chromosome seven was strongly associated with fluridone resistance (Figure 5, Table S4). The genomic window with the highest ΔSNP index (0.536) within this QTL was 29.7–31.7 Mbp. There are 256 genes within this window and a total of 344 genes within the entire 28.4–32.0 Mbp QTL region. One region of chromosome 12 (18.1–20.4 Mbp) was also above the 99th percentile; however, there is very low SNP coverage in this region (Figure 6, Table S4, see Section 4.2). A total of 52 genes are within the chromosome 12 QTL region. Two other regions (15.9–22.8 Mbp of chromosome seven and 6.2–10.9 Mbp of chromosome 13) were above the 95th percentile (Figures 6, S5–S7).

## 4 Discussion

### 4.1 A Reference Genome

We present the first reference genome assembly for Eurasian watermilfoil. The sequence is highly contiguous (21 chromosomes > 10 Mbp) and contains a high percentage (98.8%) of BUSCOs (Figure S2; Table S1). An important facet of this genome assembly is that we were able to confidently describe homoeologous chromosome groupings, which is a unique challenge when it comes to polyploid genome assembly. Being able to map future sequencing projects to subgenome‐specific locations will enable less data to be discarded from multi‐mapping issues and higher confidence in the remaining sequence data. However, the subgenome assignments between these chromosomal groups remain unknown. That is, we have yet to determine if chromosomes 1 and 4, for example, come from the same ancestor. In addition to the sequence assembly, we created a high‐quality annotation of the genome, containing 100,854 genes with 60.8% of those genes having predicted functions.

Many invasive plant species are lacking genomic resources (Matheson and McGaughran 2022). The high quality reference genome assembly of invasive Eurasian watermilfoil we present here represents one of only a few invasive aquatic plant genomes. To our knowledge, this is the first high quality reference genome assembly available for any species in the family Haloragaceae. This genome starts the sequencing of this evolutionary lineage and facilitates comparative genomic analyses in the future. As shown in this study, this reference genome is also a resource for resequencing studies in this highly managed invasive species. This genome would also likely serve as a reference in related *Myriophyllum* species.

### 4.2 Target Site Resistance

Our examinations of the *pds* gene provide no evidence for a target‐site mechanism in the fluridone resistant E_MISGP_380 Eurasian watermilfoil strain. We found no evidence for a structure‐based target site resistance mechanism in the fluridone resistant genotype E_MISGP_380. Natural variation in the *pds* gene existed across the examined genotypes with no correlation to resistance. The two identified missense mutations in the phytoene desaturase gene copies were shared between resistant and susceptible individuals. More commonly, heterozygous sites within the *pds* gene, including intronic regions, were found in the susceptible parent. This is likely due to the susceptible parent H_MYR_10199 being a hybrid between Eurasian and northern watermilfoil.

We also found no evidence for expression‐based target site resistance. We saw no differences in *pds* expression between control and treated environments or between fluridone resistant (E_MISGP_380) and susceptible (H_MISGP_457) genotypes.

### 4.3 Non‐Target Site Resistance

In this study, we developed an F2 population from a fluridone resistant (E_MISGP_380) and fluridone susceptible (H_MYR_10199) cross to identify genomic regions associated with fluridone resistance. Using QTL‐seq, we identified genomic regions significantly associated with fluridone resistance. We have strong evidence for one putative QTL on chromosome seven, based on being above the 99th percentile of the null distribution of expected ΔSNP index values. The elevated region on chromosome 12 has very low coverage of SNPs and the ΔSNP index for the region is only based on two SNPs (position 18,033,321 and 18,491,713). Because of this lack of coverage, we do not have enough evidence to declare this region as a putative QTL of the same confidence as the region on chromosome seven. Given more data and better coverage of this region, more evidence may come out supporting this region as a QTL and it is an important region of the genome to keep in mind when discussing resistance in this population. Two other regions are above the 95th percentile as well, one on chromosome seven adjacent to the previously discussed region and one on chromosome 13. It is possible the QTL on chromosome seven spans both elevated regions before and after the centromere or there may be two QTLs on chromosome seven. While the region on chromosome 13 has high SNP coverage, there is a much wider range in individual ΔSNP index values across this region, and the mean ΔSNP index values are on the edge of significance. Additionally, it appears that susceptible individuals are also enriched for alternate alleles in this region than in surrounding regions, just not to the same extent as resistant individuals (Figures S5 and S6). Because of this, we report that there is some evidence of association between SNPs in this region and fluridone resistance; however, options such as larger bulks, higher coverage, or fine‐mapping would better elucidate trait association in this region (further discussed in Section 4.3).

Altogether, our QTL‐seq results combined with a lack of differences in the *pds* gene structure or expression indicate that fluridone resistance in the E_MISGP_380 strain is due to non‐target site mechanism(s). Additionally, our RT‐qPCR gene expression experiment demonstrated that causal gene(s) within a QTL are not differentially affecting *pds* expression in the resistant genotype.

Additionally, a weed population's response to an herbicide and the herbicide's efficacy can be affected by many different physiological, ecological, and environmental factors, such as growth state, community composition, temperature, or water viscosity (Fox and Murphy 1990; Varanasi et al. 2016). Assay to assay, differences in phenotype are one of the challenges in utilizing an approach like CIM, where confident phenotype data is needed for the whole population. In current and future work, we are placing an emphasis on multiple and repeated measures of herbicide response and we have found biomass to be a robust measurement at the phenotype extremes (Hannay et al. 2024).

### 4.4 Genetic Architecture of Resistance

Although we did not identify the causal variant(s) for resistance in E_MISGP_380, our study provides some insight into the genetic architecture of resistance. We found evidence that resistance in this genotype may be due to some extent to a dominant resistance allele found on chromosome seven and that E_MISGP_380 (the resistant parent) is heterozygous for the resistance allele. We determined this by filtering out sites where the resistant parent was heterozygous from the QTL‐seq variant dataset. We then re‐ran the QTL‐seq analysis and found no evidence for the chromosome seven QTL or any other (Figure S8). Therefore, we can conclude that the resistant parent is not homozygous for the resistance allele(s) and is instead heterozygous. We can also conclude that the resistance allele(s) have a dominant effect over susceptible allele(s).

Additionally, there are other regions that approach significance in the QTL‐seq analysis (e.g., chromosome one around 42 Mb, chromosome 13 around 7 Mb). It is unknown whether further analysis would result in a stronger association between resistance variants in these regions or if the proportion of alternate alleles in the resistant bulk was elevated by random chance. These regions will be important to keep in mind when further examining the genes that contribute to resistance and whether resistance in this genotype is due to one or a few mutations.

The chromosome seven QTL region is large, but more opportunities for recombination could help narrow down this region and the putative causal variants to examine. A larger mapping population (e.g., more individuals) and/or further generations could give more chances for recombination in this region, and both are considerations for future QTL mapping studies in this system. A classic QTL mapping approach (e.g., composite interval mapping (CIM)) may also be a suitable follow‐up analysis to narrow putative QTL regions from bulk segregant analyses (Wang et al. 2022).

Lastly, it is unknown if fluridone resistance in other Eurasian watermilfoil genotypes is due to the same mechanism. Nevertheless, construction of biomarkers in the region(s) identified in this study would allow us to rapidly screen suspected fluridone resistant populations for this resistance mechanism.

### 4.5 Management Implications

Eurasian watermilfoil is a serious management concern in the United States. It is one of the most widely distributed introduced aquatic plants and is present in at least 48 U.S. states (Pfingsten et al. 2025). Although there are a handful of management tools, the most ubiquitous are herbicides. Therefore, herbicide‐resistant genotypes greatly impair our ability to manage this species. Elucidating the molecular mechanism of resistance opens the door for more efficient assays to identify resistant genotypes and minimize failed control plans, thus saving effort, time, and money.

Our results in this study indicate that fluridone resistance in the E_MISGP_380 genotype from Lake Lansing, MI, is due to at least one dominant non‐target site mutation located on chromosome seven. These findings are a huge first step towards the creation of fluridone resistance biomarkers for screening unknown watermilfoil populations using SNPs located in the QTL region. The confirmation of a dominant resistant allele in E_MISGP_380 also has implications for how resistance may continue to evolve in Eurasian watermilfoil populations and our ability to detect it using biomarkers. Biomarkers will have to be discerning enough to identify heterozygous genotypes and in close enough linkage with the causal variant for us to identify it on other genetic backgrounds.

Another important aspect of resistance is the repeatability and potential for spread. Eurasian watermilfoil is mostly clonal (see Thum et al. 2020), therefore it is unlikely for resistance mutations from this genotype to be spread to other backgrounds via sexual reproduction. However, managing and mitigating the spread of herbicide‐resistant vegetative fragments will be a critical step in preventing the spread of E_MISGP_380 into new waterways. The development and testing of resistance biomarkers on other fluridone‐resistant populations will provide more insight into the repeatability of this particular resistance mechanism. The identification of non‐target site resistance in Eurasian watermilfoil also has importance when it comes to cross‐resistance and multiple resistance. By definition, TSR mechanisms will not affect the efficacy of herbicides with different modes of action. However, NTSR can confer resistance to multiple herbicides, either within the same MOA or in entirely different MOAs (Jugulam and Shyam 2019; Delye 2013). We have evidence that this fluridone‐resistant strain (E_MISGP_380) is susceptible to 2,4‐D, a synthetic auxin herbicide (Wolfe 2021). However, we do not have any data on its response to other herbicides used in aquatic systems, such as other PDS‐inhibitors. Future studies should test the response of this genotype to other commonly used herbicides for aquatic plant management to determine if it is also resistant to other herbicides.

This study is also novel for the usage of QTL‐mapping in invasive aquatic weeds. Invasive aquatic plant management is a field that has historically had very few genetic or genomic studies when compared to terrestrial counterparts, despite the large management community and the unique life history of many aquatic macrophytes. The development and usage of QTL‐mapping in aquatics opens the door for similar analyses in other aquatic plants, for herbicide response or invasive traits, such as turion production.

## 5 Conclusion

Although Eurasian watermilfoil is the second weed species to have been confirmed as non‐target site resistant to PDS‐inhibitors (see diflufenican and fluridone resistant wild radish in Lu et al. 2020), this study is the first to identify genomic regions associated with non‐target site resistance to a PDS‐inhibitor. We hope this research encourages and paves the way for more genomics work in understudied systems and traits, even in polyploid species.

We demonstrate in this study the successful use of QTL‐seq for the identification of loci associated with fluridone resistance in Eurasian watermilfoil. We also demonstrate the successful creation and use of a high‐quality chromosome level genome assembly of the allohexaploid Eurasian watermilfoil. This reference genome will greatly facilitate future genomics work in this study system such as the creation of diagnostic biomarkers for herbicide resistance and further understanding of the genetic diversity and capacity of invasive populations.

## Conflicts of Interest

The authors declare no conflicts of interest.

## Supporting information


**Data S1:** eva70193‐sup‐0001‐Supinfo.docx.

## Data Availability

Code for the bioinformatics performed in this study is available on GitHub (https://github.com/bu20dy/Myrio‐resistance‐mechanism). qPCR data and phenotype data for the F2 population can also be found within the GitHub repository. The genome was published in the National Center for Biotechnology Information (BioSample accession SAMN52826724).
