# Correction to “Trait Mapping Utilizing a Newly Constructed Genome for Allohexaploid Invasive Eurasian Watermilfoil (*Myriophyllum spicatum*) Reveals a Non‐Target Site QTL Associated With Fluridone Resistance”

**DOI:** 10.1111/eva.70366

**Published:** 2026-11-14

Hannay, D. , G. M. Chorak , A. Harkess , et al. 2026. “Trait Mapping Utilizing a Newly Constructed Genome for Allohexaploid Invasive Eurasian Watermilfoil (*Myriophyllum spicatum*) Reveals a Non‐Target Site QTL Associated With Fluridone Resistance.” Evolutionary Applications 19, no. 1: e70193. 10.1111/eva.70193.PMC1278919141523668

In Figure 1, the legend of the inset boxplot incorrectly labeled “Resistant Bulk” as orange and “Susceptible Bulk” as blue. The legend should read “Susceptible Bulk” as orange and “Resistant Bulk” as blue. The corrected Figure 1 is provided below.

**FIGURE 1:**
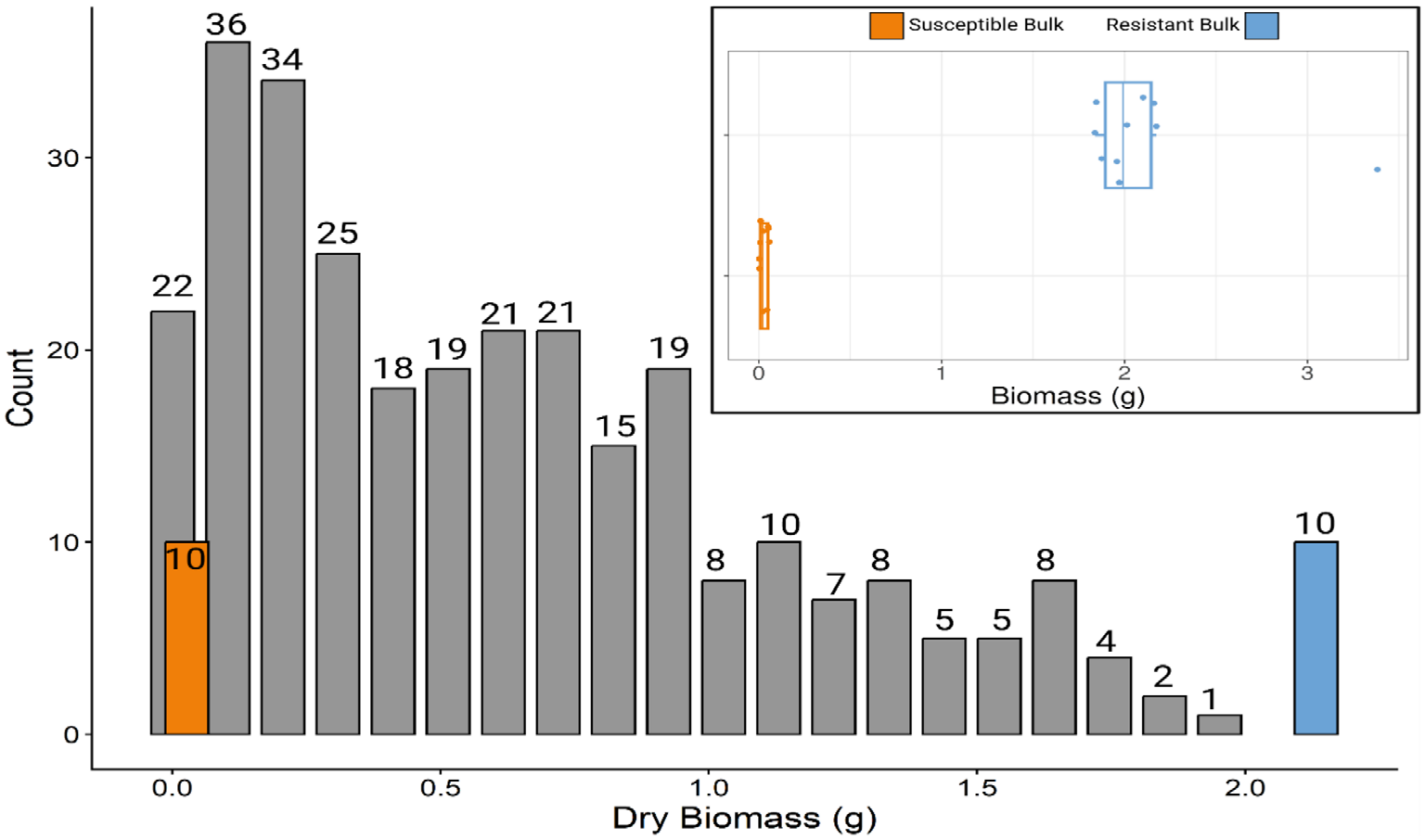
Distribution of F2 mapping population's dry biomass measurements after fluridone treatment (*n* = 308). Bars are centered around the mean for each bin. Inset boxplot shows the dry biomass measurements for the ten highly susceptible (orange) and ten highly resistant (blue) individuals sequenced for QTL analysis. Numbers above bars represent counts.

We apologize for this error.

