# Supplementary material for: Trait Mapping Utilizing a Newly Constructed Genome for Allohexaploid Invasive Eurasian Watermilfoil ( Myriophyllum spicatum ) Reveals a Non‐Target Site QTL Associated With Fluridone Resistance
Source: Evol Appl. 2026 Jan 9;19(1):e70193. doi: 10.1111/eva.70193 (PMC12789191; doi:10.1111/eva.70193)
Supplement: Supplementary file 1 — Data S1: eva70193‐sup‐0001‐Supinfo.docx. [file EVA-19-e70193-s001.docx]

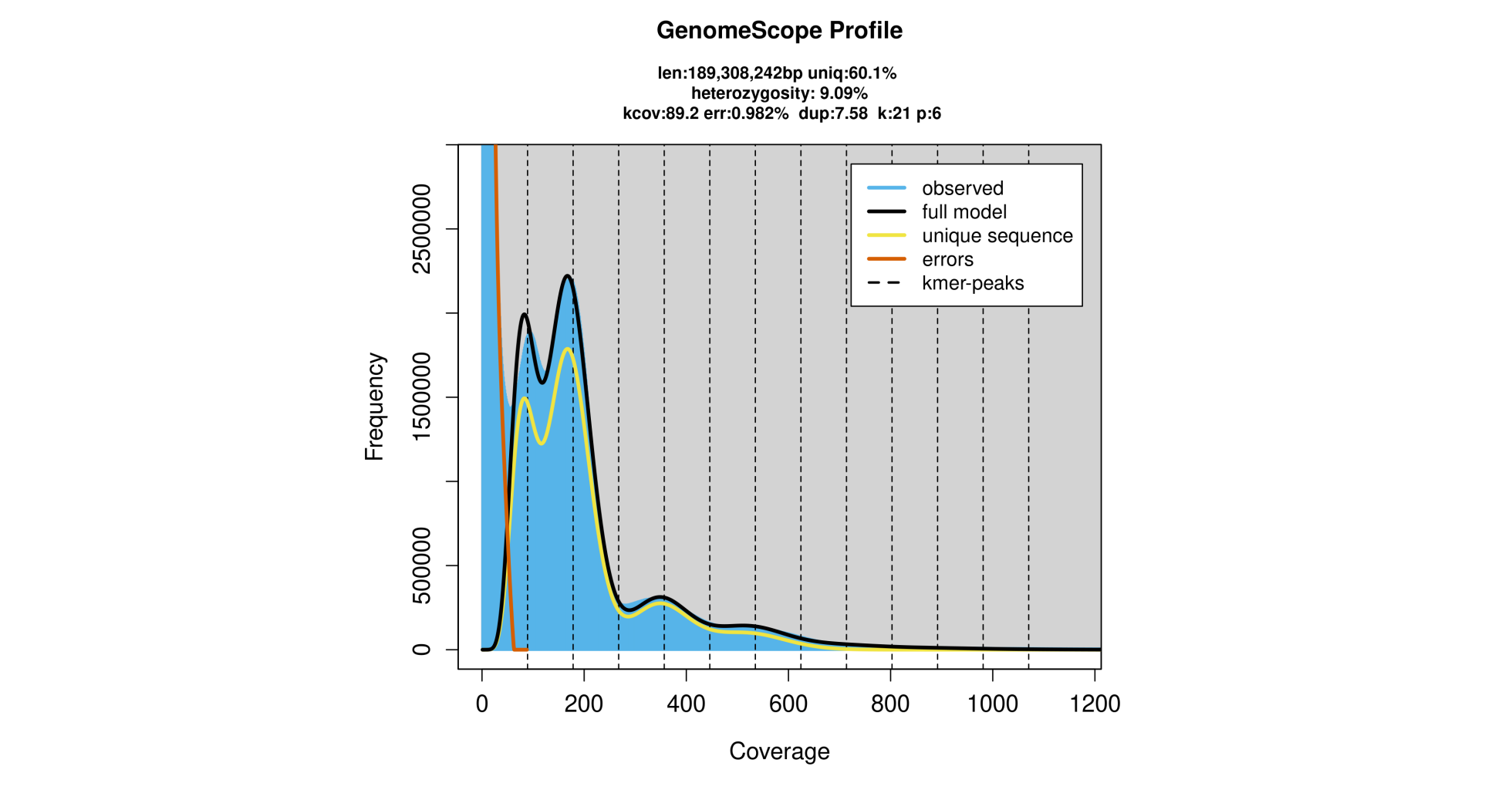


Supplemental Figure 1. GenomeScope 2.0 plot of the K-mer spectra and fitted models for the Illumina Omni-C reads generated for assembly of the *Myriophyllum spicatum* genome.


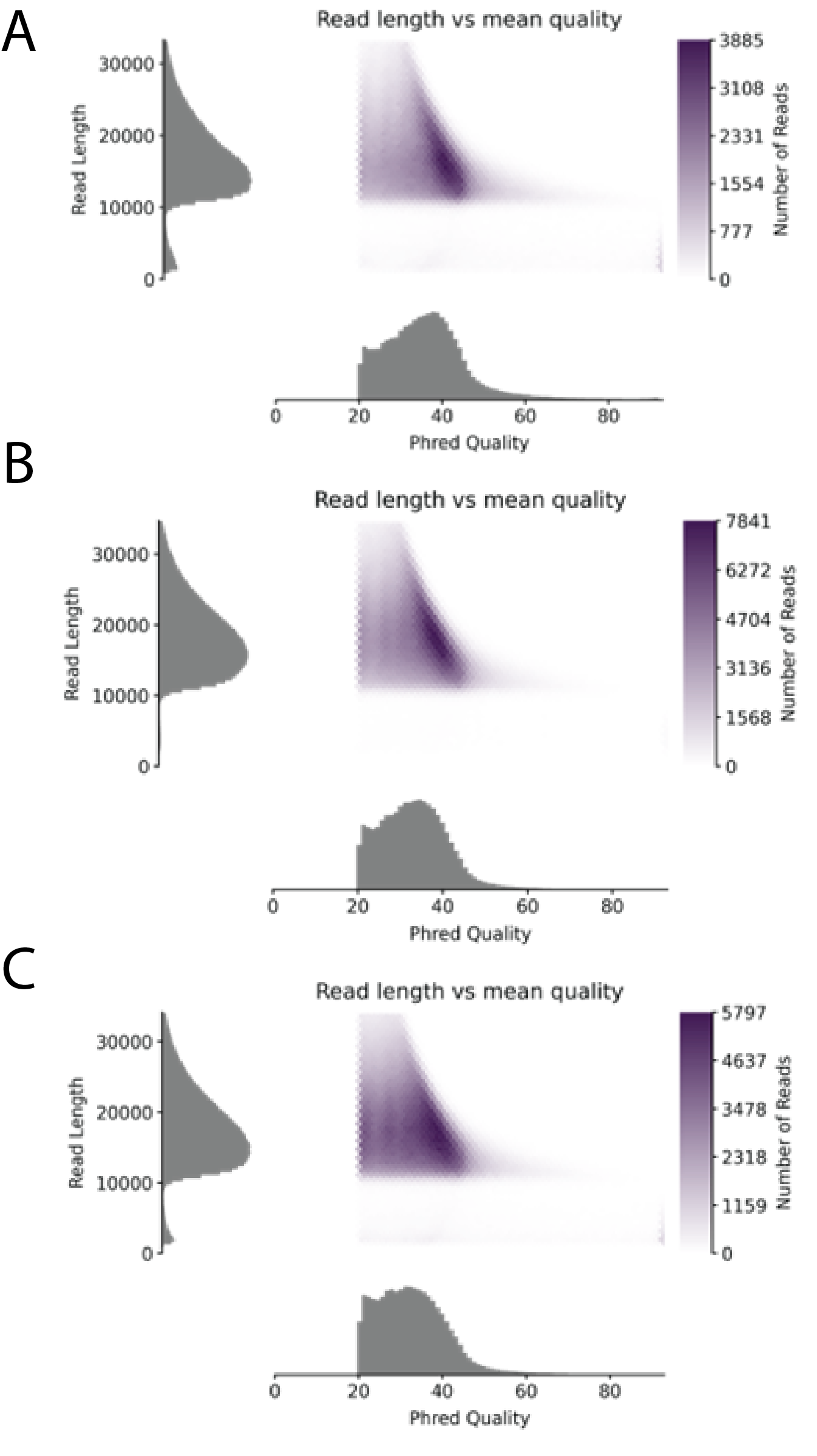


Supplemental Figure 2. A plot of raw HiFi sequencing read length and quality. Saturation of color represents the number of reads with that length and quality score. Each plot shows the raw reads from each run on the PacBio Sequel II generated for the *Myriophyllum spicatum* genome assembly.


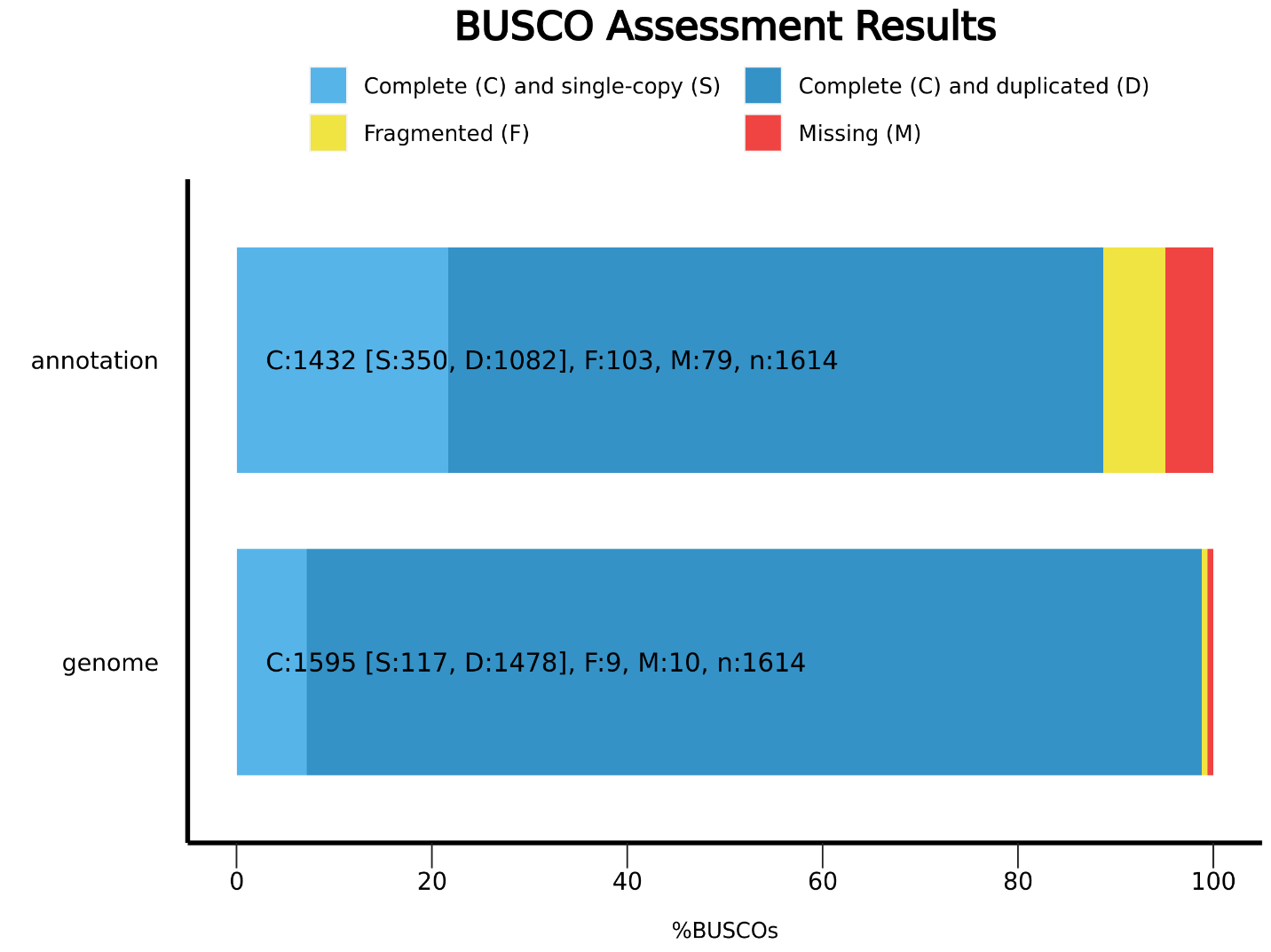
 Supplemental Figure 3. A plot showing the percentage of benchmarking universal single copy orthologs (BUSCOs) found in the genome sequence and the annotated protein sequence of the *Myriophyllum spicatum* genome.

Supplemental Figure 4. Plot of the 21 chromosome scaffolds from the *Myriophyllum spicatum* genome assembly. A change in color represents a gap of at least 100bp or a new contig in the scaffold and the numbers at the end of the scaffold shows the number contigs in that scaffold. Red asterisks indicate a significant number of telomere repeats were found in that location.


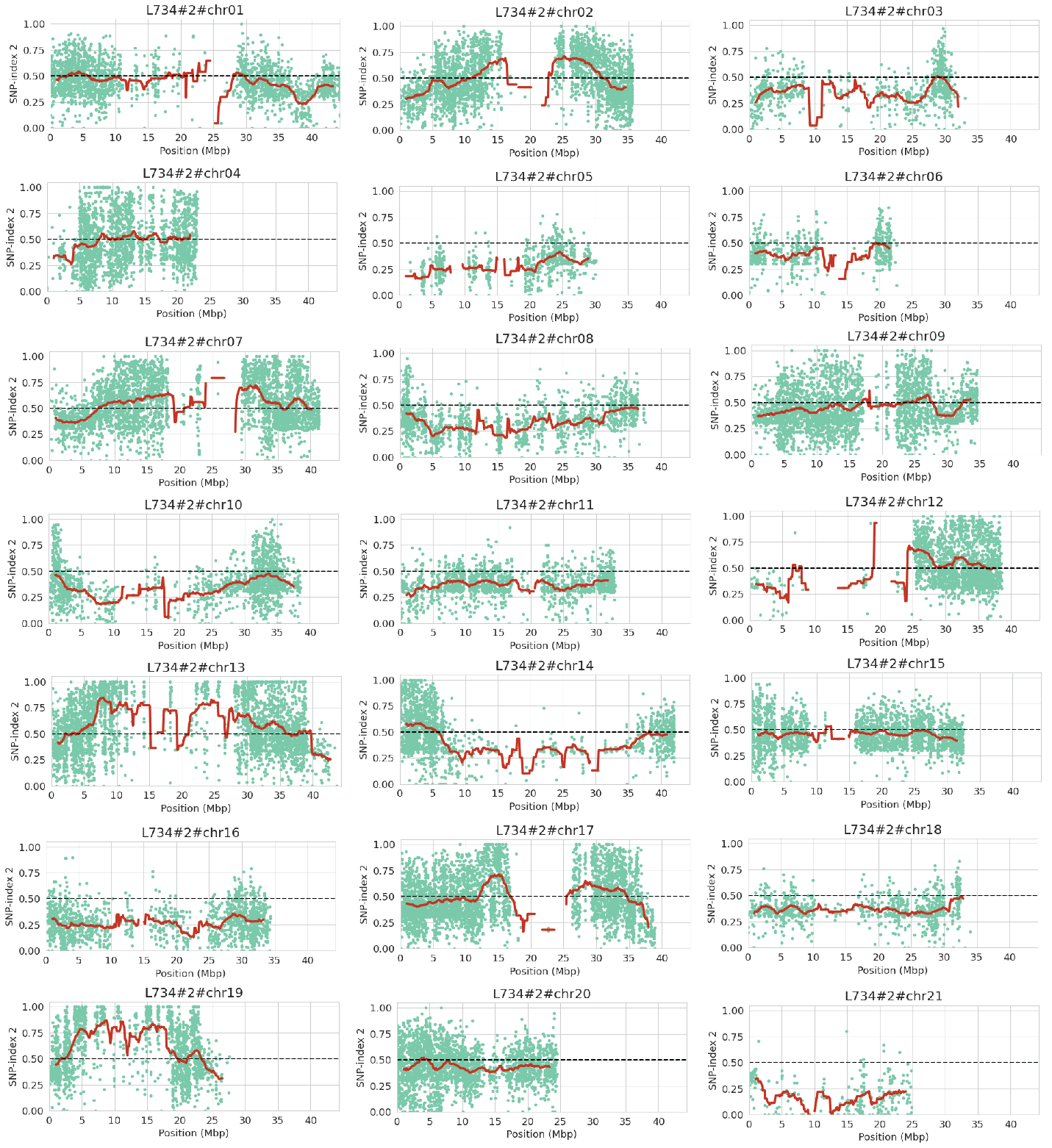


Supplemental Figure 5. Proportion of alternate alleles (SNP-indices; green dots) present in bulked variant calling of 10 fluridone resistant E_MISGP_380 x H_MYR_10199 F2 individuals. Red lines in all panels are the average calculated SNP-indices for all SNPs within a 2Mbp window with a step size of 100kbp.


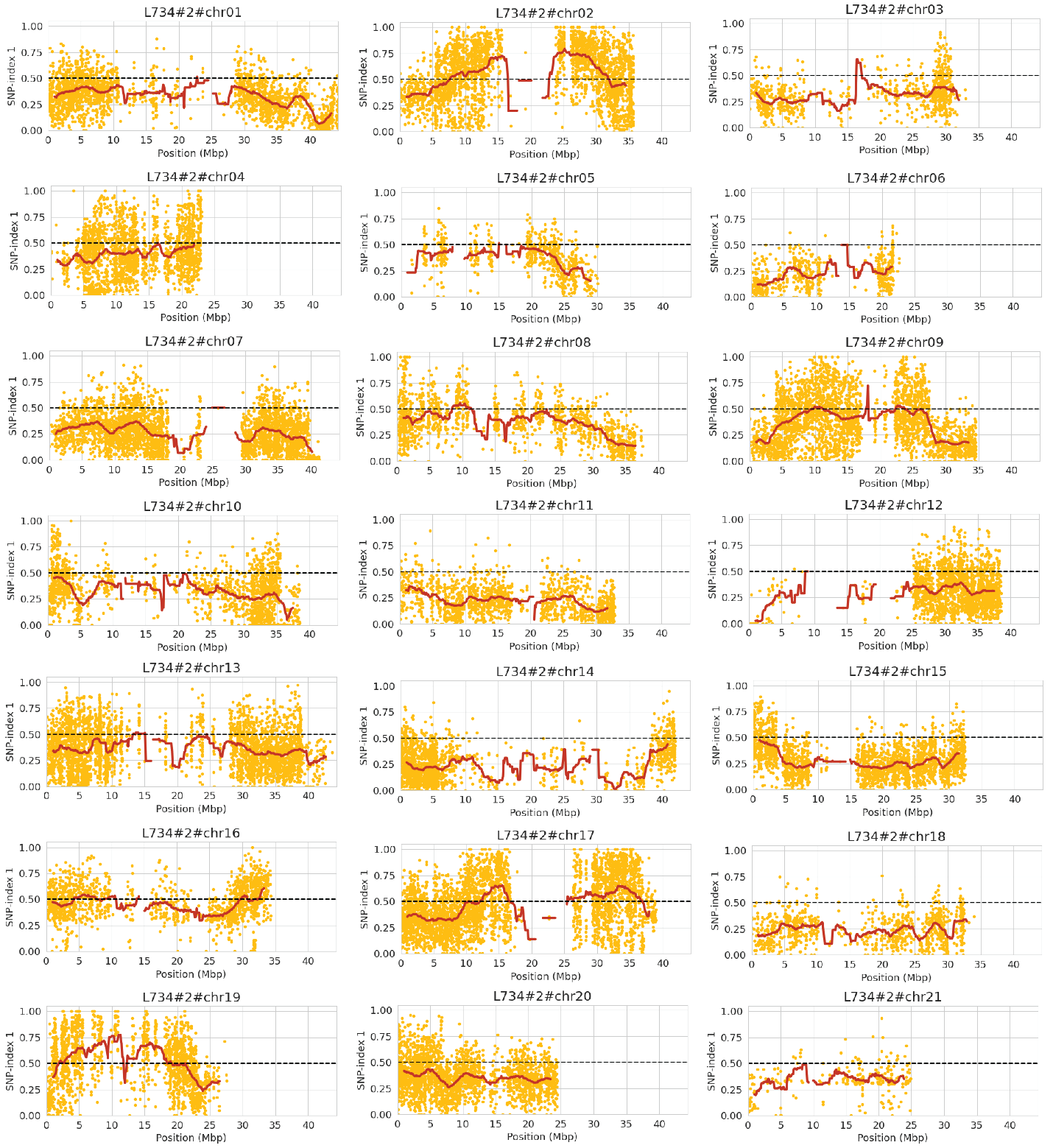


Supplemental Figure 6. Proportion of alternate alleles (SNP-indices; orange dots) present in bulked variant calling of 10 fluridone susceptible E_MISGP_380 x H_MYR_10199 F2 individuals. Red lines in all panels are the average calculated SNP-indices for all SNPs within a 2Mbp window with a step size of 100kbp.


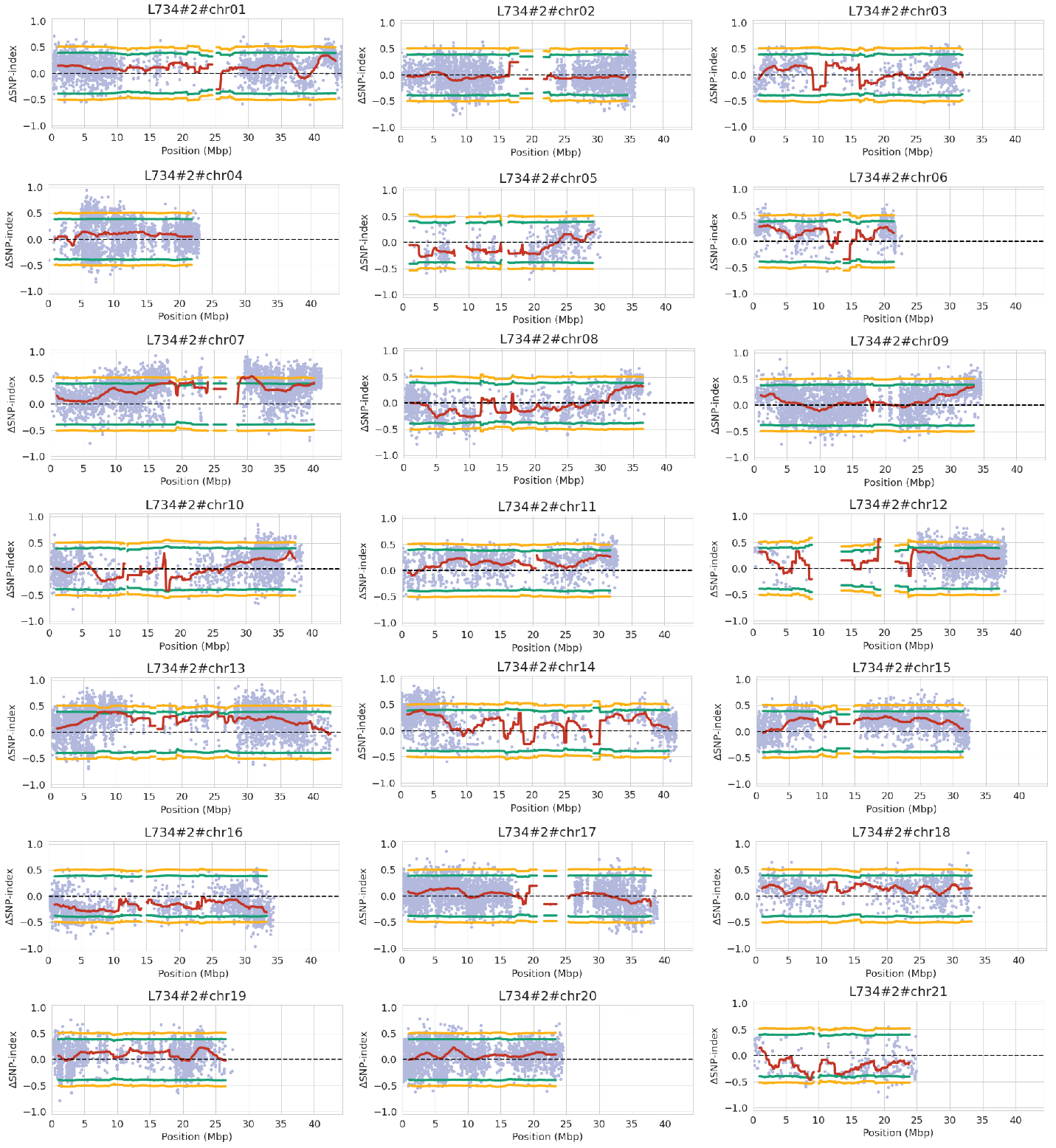


Supplemental Figure 7. Delta SNP-index values (R - S; purple dots) between 10 resistant and 10 susceptible E_MISGP_380 x H_MYR_10199 F2 individuals. Red lines in all panels are the average calculated SNP-indices for all SNPs within a 2Mbp window with a step size of 100kbp. Orange and green lines represent the 99th and 95th confidence intervals respectively for a null delta SNP-index distribution calculated from 5,000 replicated permutation tests.


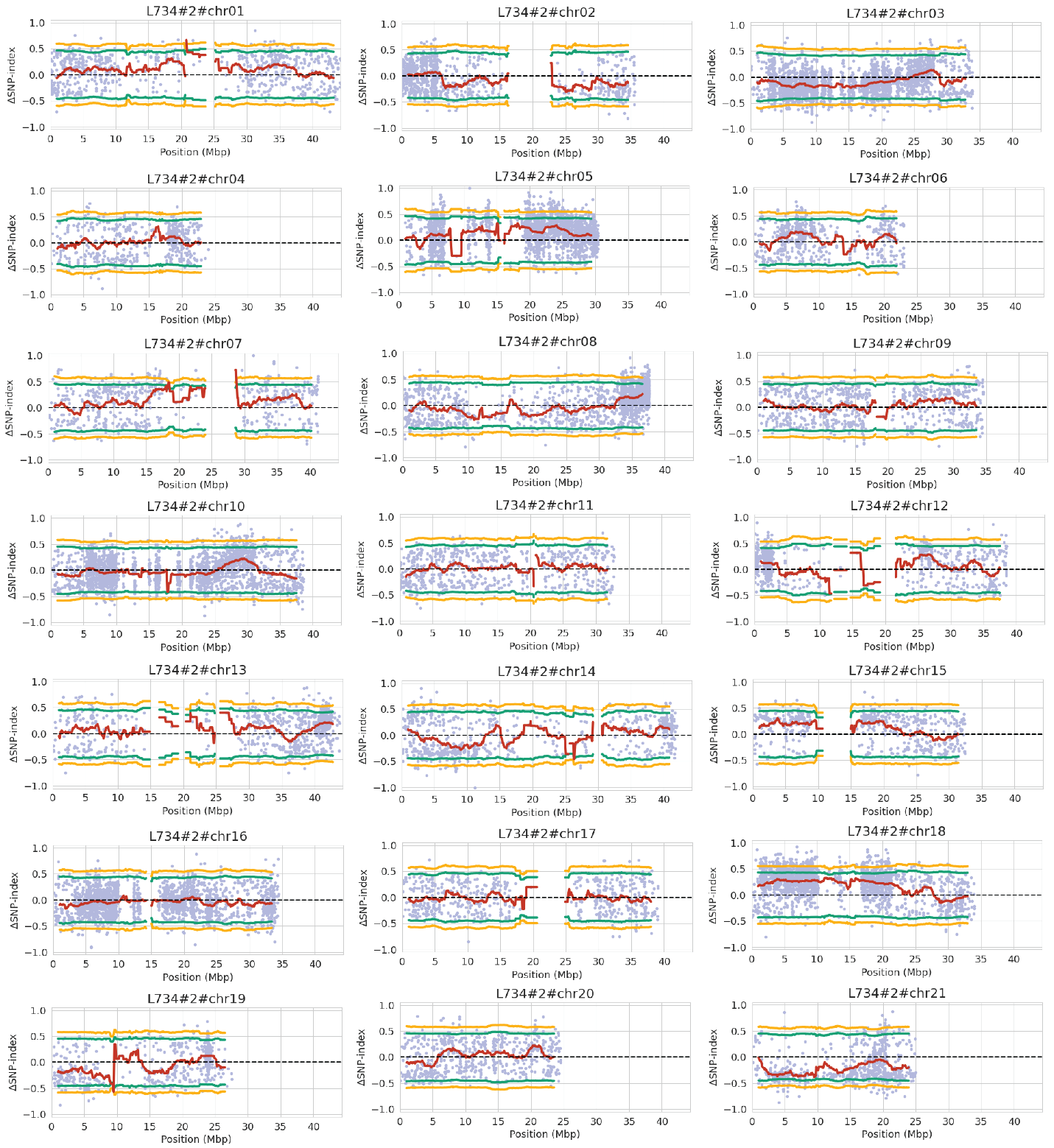


Supplemental Figure 8. QTL-seq results after filtering out sites where the resistant parent E_MISGP_380 was heterozygous. SNP-index values (R - S; purple dots) between 10 resistant and 10 susceptible E_MISGP_380 x H_MYR_10199 F2 individuals. Red lines in all panels are the average calculated SNP-indices for all SNPs within a 2Mbp window with a step size of 100kbp. Orange and green lines represent the 99th and 95th confidence intervals respectively for a null delta SNP-index distribution calculated from 5,000 replicated permutation tests.

| **Minimum Scaffold Length** | **Number of Scaffolds** | **Number of Contigs** | **Total Scaffold Length** | **Total Contig Length** | **Scaffold Contig Coverage** |
| --- | --- | --- | --- | --- | --- |
| All | 276 | 453 | 743,213,248 | 743,177,848 | 100.00% |
| 500 B | 276 | 453 | 743,213,248 | 743,177,848 | 100.00% |
| 1 KB | 276 | 453 | 743,213,248 | 743,177,848 | 100.00% |
| 2.5 KB | 272 | 449 | 743,208,248 | 743,172,848 | 100.00% |
| 5 KB | 269 | 446 | 743,198,248 | 743,162,848 | 100.00% |
| 10 KB | 268 | 445 | 743,191,982 | 743,156,582 | 100.00% |
| 25 KB | 266 | 443 | 743,154,688 | 743,119,288 | 100.00% |
| 50 KB | 170 | 347 | 739,322,188 | 739,286,788 | 100.00% |
| 100 KB | 62 | 226 | 732,117,641 | 732,084,841 | 100.00% |
| 250 KB | 31 | 175 | 727,536,163 | 727,507,363 | 100.00% |
| 500 KB | 24 | 158 | 725,497,430 | 725,470,630 | 100.00% |
| 1 MB | 22 | 154 | 724,217,600 | 724,191,200 | 100.00% |
| 2.5 MB | 22 | 154 | 724,217,600 | 724,191,200 | 100.00% |
| 5 MB | 22 | 154 | 724,217,600 | 724,191,200 | 100.00% |
| 10 MB | 21 | 74 | 718,944,429 | 718,933,829 | 100.00% |
| 25 MB | 18 | 60 | 646,982,332 | 646,973,932 | 100.00% |

Supplemental Table 1. Genome scaffold statistics

| **seq_id** | **seq_len** | **action** | **div** | **agg_cont_cov** | **top_tax_name** |
| --- | --- | --- | --- | --- | --- |
| scaffold_29 | 281192 | EXCLUDE | proteobacteria | 83 | Methylophilus medardicus |
| scaffold_40 | 178339 | EXCLUDE | proteobacteria | 66 | Methylophilus sp. |
| scaffold_67 | 96406 | EXCLUDE | proteobacteria | 81 | Aquabacterium terrae |
| scaffold_76 | 90060 | EXCLUDE | proteobacteria | 80 | Methylophilus medardicus |
| scaffold_86 | 82251 | EXCLUDE | proteobacteria | 76 | Methylotenera versatilis |
| scaffold_87 | 82003 | EXCLUDE | proteobacteria | 64 | Ideonella sakaiensis |
| scaffold_89 | 80601 | EXCLUDE | proteobacteria | 75 | Methylotenera sp. |
| scaffold_105 | 68751 | EXCLUDE | proteobacteria | 79 | Methylotenera versatilis |
| scaffold_110 | 67505 | EXCLUDE | proteobacteria | 78 | Hylemonella sp. |
| scaffold_116 | 66446 | EXCLUDE | planctomycetes | 31 | Rubinisphaera margarita |
| scaffold_117 | 66410 | EXCLUDE | proteobacteria | 58 | Methylotenera versatilis |
| scaffold_124 | 63848 | EXCLUDE | proteobacteria | 100 | Aquidulcibacter sp. |
| scaffold_136 | 60395 | EXCLUDE | proteobacteria | 74 | Methylotenera sp. |
| scaffold_145 | 58758 | EXCLUDE | proteobacteria | 76 | Ideonella benzenivorans |
| scaffold_152 | 57074 | EXCLUDE | proteobacteria | 75 | Methylotenera versatilis |
| scaffold_157 | 56173 | EXCLUDE | proteobacteria | 85 | Hylemonella sp. |
| scaffold_159 | 55768 | EXCLUDE | proteobacteria | 72 | Methylotenera versatilis |
| scaffold_164 | 54515 | EXCLUDE | proteobacteria | 83 | Methylotenera sp. |
| scaffold_168 | 54216 | EXCLUDE | proteobacteria | 95 | Methylotenera sp. |
| scaffold_182 | 51917 | EXCLUDE | rotifers | 48 | Brachionus paranguensis |
| scaffold_184 | 51407 | EXCLUDE | proteobacteria | 89 | Methylotenera oryzisoli |
| scaffold_217 | 46570 | EXCLUDE | proteobacteria | 54 | Ideonella sakaiensis |
| scaffold_228 | 44412 | EXCLUDE | proteobacteria | 81 | Methyloradius palustris |
| scaffold_231 | 43798 | EXCLUDE | proteobacteria | 100 | Piscinibacter sp. |
| scaffold_241 | 41229 | EXCLUDE | proteobacteria | 95 | Methylophilus methylotrophus |
| scaffold_248 | 39171 | EXCLUDE | proteobacteria | 89 | Methylotenera sp. |
| scaffold_250 | 39025 | EXCLUDE | proteobacteria | 85 | Methylophilus methylotrophus |
| scaffold_255 | 38161 | EXCLUDE | proteobacteria | 79 | Ideonella sakaiensis |
| scaffold_263 | 36386 | EXCLUDE | proteobacteria | 100 | Hylemonella sp. |
| scaffold_275 | 33279 | EXCLUDE | proteobacteria | 94 | Hylemonella sp. |
| scaffold_282 | 31535 | EXCLUDE | proteobacteria | 73 | Kinneretia asaccharophila |
| scaffold_287 | 29756 | EXCLUDE | proteobacteria | 88 | Aquincola rivuli |
| scaffold_305 | 2000 | EXCLUDE | proteobacteria | 99 | Ideonella sakaiensis |

Supplemental Table 2. Output of FCS-GX.

| **Variable** | **Sum of squares** | **Mean sum of squares** | **Numerator degrees of freedom** | **Denominator degrees of freedom** | **F value** | **P value** |
| --- | --- | --- | --- | --- | --- | --- |
| Genotype | 0.83566 | 0.83566 | 1 | 52.34 | 1.0304 | 0.3147 |
| Treatment | 0.03742 | 0.03742 | 1 | 52.34 | 0.0461 | 0.8308 |
| Genotype:Treatment interaction | 0.77581 | 0.77581 | 1 | 52.34 | 0.9566 | 0.3325 |

Supplemental Table 3. Analysis of variance (ANOVA) results of *pds* fold change determined via RT-qPCR. The reference gene used to calculate fold change was *otu1*.

| **Chr** | **Region** | **99^th^ percentile for mean Δ SNP index (R – S)** | **95^th^ percentile for mean Δ SNP index (R – S)** | **Mean susceptible SNP index** | **Mean resistant SNP index** | **Mean Δ SNP index (R – S)** |
| --- | --- | --- | --- | --- | --- | --- |
| 7 | 28.4 – 30.4 Mbp | 0.512 | 0.398 | 0.184 | 0.701 | 0.516 |
| 7 | 28.5 – 30.5 Mbp | 0.512 | 0.397 | 0.182 | 0.701 | 0.519 |
| 7 | 29.5 – 31.5 Mbp | 0.511 | 0.396 | 0.180 | 0.702 | 0.522 |
| 7 | 29.6 – 31.6 Mbp | 0.509 | 0.395 | 0.176 | 0.709 | 0.533 |
| 7 | 29.7 – 31.7 Mbp | 0.508 | 0.394 | 0.177 | 0.713 | 0.536 |
| 7 | 29.8 – 31.8 Mbp | 0.508 | 0.394 | 0.183 | 0.715 | 0.533 |
| 7 | 29.9 – 31.9 Mbp | 0.508 | 0.394 | 0.185 | 0.713 | 0.528 |
| 7 | 30.0 – 32.0 Mbp | 0.507 | 0.394 | 0.191 | 0.709 | 0.519 |
| 12 | 18.1 – 20.1 Mbp | 0.525 | 0.408 | 0.375 | 0.933 | 0.558 |
| 12 | 18.2 – 20.2 Mbp | 0.525 | 0.408 | 0.375 | 0.933 | 0.558 |
| 12 | 18.3 – 20.3 Mbp | 0.525 | 0.408 | 0.375 | 0.933 | 0.558 |
| 12 | 18.4 – 20.4 Mbp | 0.525 | 0.408 | 0.375 | 0.933 | 0.558 |

Supplemental Table 4. All 2 Mbp sliding window genomic regions above the 99^th^ percentile of the null distribution from the QTL-seq analysis.
